# Supersaturation-Dependent Competition between β and κ Phases in the MOVPE Growth of Ga_2_O_3_ on Al_2_O_3_ (0001) and GaN (0001) Substrates

**DOI:** 10.1021/acsami.5c13401

**Published:** 2025-10-31

**Authors:** L. Seravalli, A. Ugolotti, R. Bergamaschini, M. Bosi, I. Cora, F. Mezzadri, P. Mazzolini, L. Cademartiri, I. Bertoni, Zs. Fogarassy, B. Pécz, O. Bierwagen, A. Ardenghi, S. Leone, L. Nasi, L. Miglio, R. Fornari

**Affiliations:** ‡ CNR-IMEM (Institute of Materials for Electronics and Magnetism), 43124 Parma, Italy; § Department of Mathematical, Physical and Computer Sciences, 9370 University of Parma, 43124 Parma, Italy; ⊥ Department of Chemistry, Life Sciences, Environmental Sustainability, University of Parma, 43124 Parma, Italy; ∥ 226378 HUN_REN Centre for Energy Research, Institute for Technical Physics and Materials Science, 1121 Budapest, Hungary; ¶ Department of Materials Science, 9305 University of Milano−Bicocca, 20125 Milano, Italy; # Paul-Drude-Institut für Festkörperelektronik, 28411 Leibniz-Institut im Forschungsverbund Berlin eV, Hausvogteiplatz 5−7, 10117 Berlin, Germany; ∇ 28429 Fraunhofer Institute for Applied Solid State Physics, Tullastrasse 72, 79108 Freiburg, Germany

**Keywords:** gallium oxide, MOVPE, interface, XRD analysis, HRTEM, phase stabilization

## Abstract

We present an analysis of the phase stabilization of β-Ga_2_O_3_ and κ-Ga_2_O_3_ grown by metal–organic vapor-phase epitaxy with varying supersaturation of the gas-phase precursors on c-sapphire and GaN substrates. We compare in-depth structural analyses of the bulk and interface layers, also through the measurement of strain relaxation across the structure, with a comprehensive nucleation model, based on multiscale simulations. A coherent and quantitative interpretation of different stages of the nucleation of Ga_2_O_3_ phases on sapphire is elaborated on, highlighting the crucial role of the supersaturation of gas precursors and of the residual misfit strain within the initial layers in the stabilization of competing Ga_2_O_3_ polymorphs.

## Graphical abstract

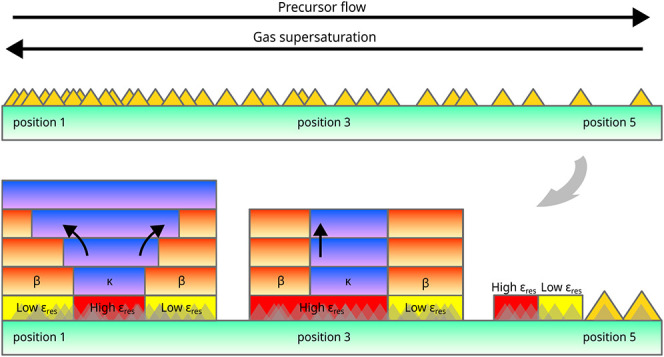

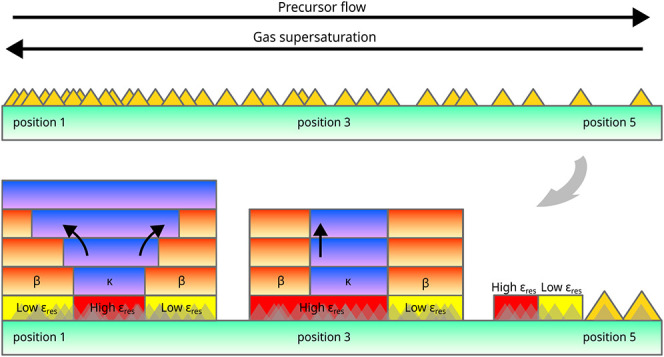

## 1 Introduction

The interest toward gallium oxide (Ga_2_O_3_) has significantly increased in the past decade because of its potential for the next generation of high power electronic devices and for novel UV-C solar-blind detectors.^1,2^ Ga_2_O_3_ crystallizes in different polymorphs (α, β, ε/κ, γ, and δ): the stable crystal structure is the monoclinic β one, that is by far the most studied. In fact, thanks to the possibility to obtain bulk β-Ga_2_O_3_ ingots from the melt, it was possible to develop different device architectures based on homoepitaxy. However, due to the intrinsic low symmetry of the monoclinic cell, β-Ga_2_O_3_ presents challenges even in homoepitaxial growth,^3^ e.g., anisotropic thermal conductivity,^4^ as well as the tendency toward cleavage.^5^

The β-Ga_2_O_3_ heteroepitaxy on sapphire (0001), results in (−201)-oriented layers characterized by the presence of six rotational domains that evolve in height with a quite disordered morphology due to the presence of the monoclinic angle.^6–8^ For these reasons, the epitaxial deposition on sapphire (0001) of metastable Ga_2_O_3_ polymorphs with higher crystallographic symmetry has become attractive. Among those , α-Ga_2_O_3_, isomorph to sapphire, that can be obtained with no rotational domains and a higher band gap (5.3 eV), and the (001) κ orthorhombic phase. It is important to notice that this phase was previously considered as hexagonal and named ε phase, hence often indicated as ε/ κ in the literature.^9^ This is now an undisputed issue, in this article we refer to this phase just as κ. One peculiar aspect of κ-Ga_2_O_3_ is its spontaneous polarization along the (001) direction, predicted to be ∼23–26 μC/cm^2^,^10^ which should allow for the confinement of high mobility two-dimensional electron gases at κ-Ga_2_O_3_/Al_*x*_Ga_2–*x*_O_3_ heterointerfaces. The carrier densities calculated for these structures (*n* ≈ 10^14^ cm^–2^) are higher than the ones obtainable in β-based heterostructures, in principle with no need for modulation doping.^11–14^

Epitaxial stabilization of Ga_2_O_3_ polymorphs has been successfully achieved on sapphire and other substrates by various techniques, such as metal–organic vapor-phase epitaxy (MOVPE),^15–18^ halide vapor-phase epitaxy (HVPE),^19–21^ molecular-beam epitaxy (MBE),^18,22^ and mist chemical vapor deposition (mist-CVD).^23–26^ Well-assessed deposition protocols are now widely available.^27^ In a previous work, it was shown that the supersaturation (i.e., the growth rate) in MOVPE with trimethylgallium (TMGa) and water precursors has a main role in controlling the nucleation of a particular Ga_2_O_3_ polymorph—even on the same substrate—and that, by reducing it, it is still possible to obtain the β-Ga_2_O_3_ phase even at temperatures of 610 and 650 °C, much lower than what previously reported in the literature for MOVPE experiments.^15^ Hence, it is possible to control the nucleation of pure β-Ga_2_O_3_, or a mix of β-Ga_2_O_3_ and κ-Ga_2_O_3_, or eventually obtain just a pure κ-Ga_2_O_3_ film, by increasing the TMGa supersaturation. Similar results were obtained by Jiang et al. using triethylgallium and O_2_ at 530 °C.^16^ Nonetheless, the reason why the κ phase grows as a compact film on top of an initial corrugated interlayer, e.g., consisting of (−201)-oriented β-Ga_2_O_3_, possibly with γ-Ga_2_O_3_ inclusions and not directly on sapphire (0001), has not been properly addressed in literature yet.

It is known that the crystal structure of the substrate is a crucial factor for the stabilization of a pseudomorphic phase in epitaxy. In particular, it was recently argued that “*the Ga*_2_ *O*_3_ *layers grown* (in a metastable phase) *are effectively phase locked with the underlying crystal structure*”,^28^ showing how in mist-CVD the growth of α-Ga_2_O_3_ may be favored on sapphire against the growth of β-Ga_2_O_3_ as the former has the same corundum crystal structure of the substrate. In a very recent paper,^29^ the origin of such phase-locking has been assessed on the basis of first-principles calculations of interface and surface energies and 2D layer nucleation modeling, showing that the formation energy of the κ phase is in between the lower one of the α phase and the higher one of the β phase.

Actually, the growth of metastable κ-Ga_2_O_3_ has also been reported in mist-CVD experiments on c-sapphire developing on top of an ultrathin interlayer of other polymorphs,^26^ and in a few HVPE experiments, both on the clean c-sapphire substrate^19^ and on top of an ultrathin GaN interlayer deposited on it,^20^ or using HCl-added MOVPE,^17^ possibly favored by the catalytic action of the Cl atoms. The stabilization of κ-Ga_2_O_3_ by physical vapor deposition means, like MBE or pulsed-laser deposition, strictly requires the use of a catalyst element (i.e., In and Sn) in a process called metal-exchange catalysis.^22,30,31^ Actually, when grown over (0001) sapphire, κ-Ga_2_O_3_ is composed of straight-tall orthorhombic domains, rotated by 120° about the (001) axis, on the hexagonal network of O atoms, appearing at both the α(0001) and β(−201) planes, thus forming a pseudohexagonal structure.^9,32^

In this already articulated landscape, the presence of other, more defective Ga_2_O_3_ phases must not be neglected. Among them, the γ polymorph, characterized by defective spinel structure with partially occupied sites, is believed to play a role in the growth of the different Ga_2_O_3_ phases, given its close relationship with the structure of the β phase, with which it shares the face-centered-cubic sublattice of O atoms.^33^ The crystallization of such stoichiometric cubic phase was achieved in postgrowth annealing of amorphous Ga_2_O_3_ films grown on c-sapphire through MBE^34^ or oxygen-reactive electron-beam evaporation.^35^ Similar phase transformations were observed in films of nominally pure κ phase grown on the same substrate through MOVPE.^36^ Indeed, the presence of γ-Ga_2_O_3_ during the epitaxial growth has been already reported in the case of MOVPE depositions,^9,16,32^ but its role on the subsequent stabilization of the κ phase on top was never addressed.

First-principles calculations of strain-free bulk cohesion energies confirm that β-Ga_2_O_3_ is the most stable polymorph,^1^ followed by κ and α phases, whose energy separation is much smaller and still debated in the literature^37,38^ Then, the γ-Ga_2_O_3_ is predicted at even higher energy.^1,39^ However, such a ranking does not explain their observed nontrivial competition during epitaxy. Therefore, there is strong interest in assessing the forces driving the growth mode of κ-Ga_2_O_3_ with respect to the β phase, in order to (i) get a deeper understanding of the stabilization of different polymorphs, (ii) control the nucleation and stabilization of the phase of interest in c-sapphire, and (iii) understand the role of the interface (and inherent strain) between the substrate and film.

In this work, we analyze the interplay between thermodynamics and elastic energy and surface/interface energies, calculated from first principles, underlying such a complex phenomenology, and we relate the phase competition to the growth conditions, namely, the supersaturation of gas-phase precursors. To this goal, we mainly focus our analysis on the experimental cases already reported in ref 15, where H_2_O and TMGa were used to grow Ga_2_O_3_ by MOVPE at 610 and 650 °C on c-sapphire, encompassing a significant variation of growth regimes. A comparison with new samples grown on wurtzite GaN (0001) is also considered to assess the role of a different substrate. A detailed description of such growth experiments is reported in Section 2.

An extensive characterization of the structural properties of the epitaxial Ga_2_O_3_ films was performed by using both X-ray diffraction (XRD) and transmission electron microscopy (TEM) imaging with geometrical phase analysis (GPA). These methods are detailed in Section 2. In particular, the interface region between the sapphire substrate and the Ga_2_O_3_ epilayer is characterized with atomic scale resolution to identify the different phases and domains in the grown layers as well as the possible formation of inclusions of β-Ga_2_O_3_ in κ/β mixed-phase material. Moreover, GPA also makes possible to quantitatively evaluate the strain relaxation across the structure, revealing that the metastable κ-Ga_2_O_3_ phase is substantially strain-free. Such results are illustrated in Section 3.

These new experimental data, combined with theoretical estimates^29,38^ of the thermodynamic parameters (formation energies, molar volumes, surface and interface energies, elastic constants, relative lattice misfits, etc.) for the α-, β-, and κ-Ga_2_O_3_ phases, allowed us to develop a comprehensive nucleation model, which will be discussed in Section 4.

Finally, in Section 5, both experimental and theoretical insights will be combined in a coherent framework, unveiling an unexpected sequence of nucleation steps of Ga_2_O_3_ phases on c-sapphire. On this quantitative basis, some additional considerations will be made on the slightly different experimental results of competitive nucleation when the substrate is wurtzite GaN (0001), focusing on the role of the different misfit strain.

## 2 Experimental Methods

### 2.1 MOVPE Growth

The epitaxial layers were deposited by MOVPE using ultrapure H_2_O and TMGa as precursors, stored in stainless steel bubblers at 30 °C and 1 °C, respectively. A flow of 400 sccm of helium was used as a carrier gas. The substrate temperature was fixed at 650 °C, the chamber pressure was 100 mbar, and the film deposition time was varied between 20 and 120 min.

The growth was carried out in a horizontal reactor, and half of a 2 in. substrate was placed on a nonrotating graphite substrate holder, in order to promote a nonuniform growth along the sample, as sketched in Figure 1. COMSOL models of the reactor and measurements with the thermocouple indicate that the temperature is homogeneous in the range of ±5 °C over the entire substrate pocket.^15^

**Figure 1:**
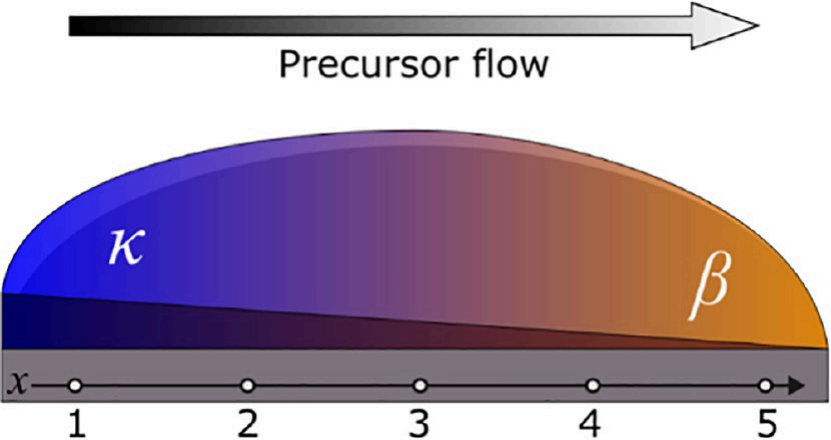
Schematic representation in the 3D perspective view of a sample, highlighting the inhomogeneous growth conditions across its diameter, from the inlet region (point 1) to the outlet/exhaust (point 5), resulting in a thickness gradient and change of the Ga_2_O_3_ phase. Numbers 1–5 correspond to evenly spaced positions along the diameter where characterizations were performed.

This enables to inspect at once, on the same sample, different growth conditions just looking at different positions across the precursor flow direction, here labeled from 1 (inlet) to 5 (outlet). The optimal MOVPE growth conditions on sapphire (sample A) were chosen on the basis of the parameters described in ref. 15, where a pure κ-Ga_2_O_3_ film was grown on sapphire at the inlet side of the sample (point 1), while a pure β phase was obtained toward the outlet/exhaust (point 5). A mixed β/κ phase Ga_2_O_3_ was observed at the intermediate positions. As discussed in ref.^15^ these conditions allowed us to achieve the best crystal quality of the κ-Ga_2_O_3_ material. The deposition time for sample A was 120 min. With the aim of providing an assessment of the major factors driving the stabilization of the different Ga_2_O_3_ phases, we performed the growth of three additional samples, labeled B–D. In sample B, we kept the same growth conditions as those for sample A, but we performed the growth on a (0001)-oriented GaN/sapphire substrate, in order to evaluate the role of the misfit strain. In the case of sample C, the growth was performed on sapphire with a deposition time reduced to 20 min. Finally, sample D was grown starting from a high-quality, ∼20-nm-thick, (−201)-oriented β-Ga_2_O_3_ layer, previously deposited by MBE on a c-plane sapphire substrate (thickness determined by X-ray reflectivity). Such a sample is meant to investigate how the crystal quality/surface roughness (root mean square ≤ 0.6 nm) of a well-defined nucleation layer could influence the subsequent MOVPE growth. The growth conditions of this sample were chosen to obtain pure κ-Ga_2_O_3_ material only, as the focus of the investigation was just the interface between epilayer and substrate, as discussed below.

The details of the growth of this β-Ga_2_O_3_ template layer are reported in Section 2.2, while characterization data are reported in Section 1 of the Supporting Information.

The thickness of the films was measured by optical reflectance in the 270–1600 nm range, using a Jasco V-770 spectrophotometer, with about 10% uncertainty. The film thickness was calculated considering a refractive index *n*(λ) obtained by ellipsometry: 0.6585 exp­(−2.759 × 10^–3^λ) + 1.75 exp­(9.837 × 10^–5^λ), where λ is the wavelength in nanometers.

The epitaxial growth of GaN was performed in a MOVPE reactor on Al_2_O_3_ (0001) substrates (having a 0.3° miscut toward the m-plane) using TMGa and NH_3_ as precursors and H_2_ as the carrier gas at a reduced pressure of 300 mbar. GaN layers were deposited with a V/III ratio of 1500 and at a wafer surface temperature of 1050 °C, as described elsewhere.^12^

### 2.2 MBE Growth

The ∼20-nm-thick MBE β-Ga_2_O_3_(−201) layer was grown in a plasma-assisted molecular-beam epitaxy (PA-MBE) chamber equipped with a radio-frequency (RF) plasma source (SPECS PCS). The deposition was performed on a 2 in. Al_2_O_3_ (0001) wafer. Prior to the growth the substrate was sequentially cleaned using acetone and isopropyl alcohol for 5 min with sonication, followed by 1 min of O_2_ plasma treatment (300 W, 1 sccm) in the growth chamber at the same substrate temperature used for the growth (580 °C). The β-Ga_2_O_3_ template was grown for 12 min by simultaneously supplying elemental gallium from a double-filament effusion cell, with a corresponding beam equivalent pressure (BEP, measured by a nude ion gauge at the substrate position) of BEP_Ga_ = 1.3 × 10^–7^ mbar and 0.33 standard cubic centimeter per minute (sccm) of oxygen (O_2_) at 300 W of RF plasma power. In Figure S1, we provide XRD and atomic force microscopy (AFM) characterization.

### 2.3 XRD Characterization

XRD data were collected using a Rigaku Smartlab XE diffractometer. Measurements were carried out with Cu Kα wavelength making use of HyPix3000 detector operated in 1D mode. The incident beam was shaped by the use of slits to approximately 1 × 2 mm^2^ at 2θ ≈ 60°. For the determination of the lattice parameters, an Euler cradle was used, allowing one to measure the 2θ position of the peaks after a thorough centering performed by a sequence of ω, φ, χ, and 2θ centerings. A series of 12 isolated peaks were selected in order to have sufficient statistics over the *h*, *k*, and *l* indices.

The computational method to obtain lattice parameters is based on a least-squares fitting of unit cell parameters, as obtained from the XRD of oriented thin films, and is described in the more depth (including error limits) in Section 2 of the Supporting Information, including Table S1 and Figure S2.

### 2.4 TEM and GPA Characterization

TEM experimental data were collected in a spherical aberration-corrected Titan Themis G2 200 (Thermo Fisher Scientific) (S)­TEM instrument equipped with a X-FEG gun operating at 200 keV and 4k × 4k CETA16 CMOS camera (Thermo Fisher Scientific). TEM lamella were prepared with a ThermoFisher Scios 2 dual-beam microscope (Eindhoven, The Netherlands) with EasyLift nanomanipulator.

The obtained high-resolution TEM (HRTEM) images were analyzed by GPA^40,41^ to quantitatively determine the strain field. Phase images were calculated from the raw HRTEM image using selected reflections on the fast Fourier transform (FFT) pattern of the HRTEM image in comparison to a reference area in the substrate. This procedure allows us to obtain deformation values (with respect to the substrate) and to follow the changes in the crystal lattices inside the epilayers along both the out-of-plane and in-plane directions of the TEM lamella of our samples at the nanometer scale.

## 3 Results and Discussion

In order to assess how the different Ga_2_O_3_ phases compete as a consequence of the inhomogeneous conditions across the reactor, the overall composition and texture of the film for samples A–C were characterized through XRD and TEM (Section 3.1) at the different positions 1–5 of the samples. Instead, the interface layer was investigated in detail through HRTEM imaging for samples C and D (Section 3.2). Finally, the impact of the initial interface layer is investigated indirectly through GPA, imaging the local strain in different areas and its correlation with the presence of structural defects at earlier stages of the deposition (Section 3.3).

### 3.1 Film Composition and Grain Orientation for Different Substrates

In Figure 2, we present a comparison of XRD patterns for samples grown on sapphire and on GaN (samples A and B), examined at positions 1, 3, and 5. The well-known diffraction peaks relative to the different phases can be clearly distinguished: 2θ = 59.87° for the (006) reflection of κ-Ga_2_O_3_ and 2θ = 59.2° for the (−603) reflection of β-Ga_2_O_3_, with the latter having a larger full-width at half-maximum due to a lower crystallinity because the growth temperature was well below the optimal one for this phase by MOVPE.

**Figure 2:**
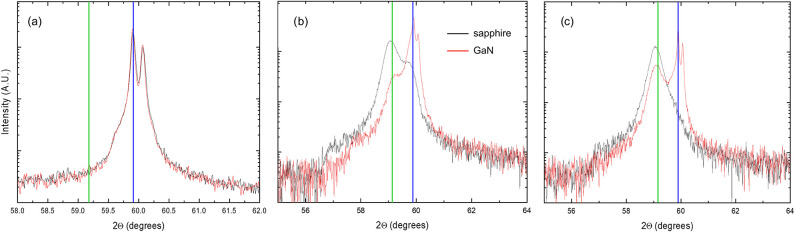
XRD patterns for sample A grown on c-sapphire (black line) and sample B on GaN (red line) at positions 1 (a), 3 (b), and 5 (c). The vertical lines represent the characteristic diffraction angles for the κ (blue) or β (green) Ga_2_O_3_ phase.

XRD data demonstrate that, for two depositions at the same temperature and growth rate but on two different substrates, the mixed-phase material grown on GaN appears to be richer in κ-Ga_2_O_3_, and a single β phase is never obtained, i.e., not even for the lowest growth rate. XRD was carried out also on sample C (position 1) and on sample D (presented in Figure S2), confirming that these samples are pure κ in the bulk, according to the XRD sensitivity.

To gain a deeper understanding of the properties of the mixed-phase material, an advanced structural analysis based on the XRD data was performed, as detailed in the Supporting Information. The estimation of the *c* lattice parameter of κ-Ga_2_O_3_ based on such analysis allows us to conclude that some distortion of the orthorhombic lattice is induced by the presence of the β-Ga_2_O_3_ material in the mixed-phase parts of the deposition. A similar increase of the *c* lattice parameter was reported for structures grown at 500 °C,^31^ being attributed to a possible influence of an additional β phase to the crystallinity. This could hint to a process where the strain compensation occurs mainly within the interlayer and that the κ-Ga_2_O_3_ grows substantially relaxed, albeit with the well-known rotational domains.

In order to get a gather additional insights about (i) the cross-section distribution of the mixed κ/β material and (ii) the properties of the interface, TEM analysis of the samples was carried out. Also, the information provided by TEM characterization was employed for the theoretical modeling of the phase stabilization, to be discussed later.

TEM analysis was carried out at point 3 of sample A (grown on sapphire) and at point 5 of sample B (grown on GaN). Interestingly, at both of these positions about 50% of the film composition is attributed to β-Ga_2_O_3_ according to XRD analysis. As the growth conditions (and the resulting β/κ ratio) of the two samples were the same, a different position in samples A and B, related to different growth rate and/or supersaturation of TMGa in the growth chamber along the precursor flow direction, should compensate for the different contributions due to the different substrates.

Large-area bright-field TEM images of the samples, reported in Figure 3, evidence β-Ga_2_O_3_ grains elongated perpendicular to the surface (see Figures S3 and S4 for images taken with a large field of view). κ-Ga_2_O_3_ appears to be more present in the first-to-grow part of the layer as straight stripes perpendicular to the surface, associated with the well-known rotational domains, while the amount of β-Ga_2_O_3_ seems to be higher closer to the surface of the sample.

**Figure 3:**
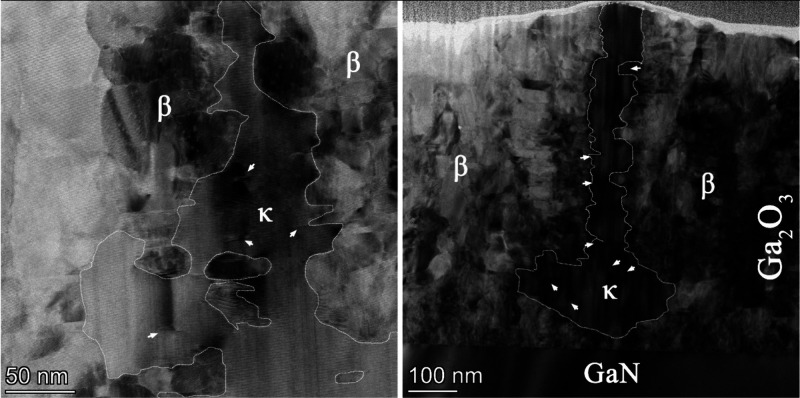
Bright-field TEM images with phase contrast of sample A on sapphire (left) and of sample B on GaN (right). The grain boundaries between the different phases have been highlighted, while white arrows point to areas where β-Ga_2_O_3_ starts to nucleate parallel to the growth of κ-Ga_2_O_3_.

Focusing on the texture of the κ/β layer in Figure 3, we highlighted the boundaries between the two phases. The domain and antiphase boundaries, which characterize the β phase, do not usually continue inside κ-Ga_2_O_3_, leading to the conclusion that β-Ga_2_O_3_ grains form during MOVPE growth alongside the κ column and not as a product from κ-Ga_2_O_3_ conversion. If β-Ga_2_O_3_ was resulting from κ-Ga_2_O_3_ conversion, grains would cut through the boundaries of κ-Ga_2_O_3_ domains. Indeed, in some parts of the sample, we found β-Ga_2_O_3_ grains that cut through a domain of κ-Ga_2_O_3_; however, we consider this possibility unlikely because it has been observed that the κ phase converts to β at much higher temperatures.^42,43^ Generally speaking, no boundary of any phase is observed to expand prominently at the expense of the other; nonetheless, we found some small, darker areas within the existing κ-Ga_2_O_3_ material, which we can identify as β-Ga_2_O_3_ grains (highlighted by white arrows in Figure 3). These grains are isolated within the matrix of the κ phase, suggesting a tight competition between the nucleation of β- and κ-Ga_2_O_3_. Indeed, the TEM image taken for sample A on sapphire shows a more inhomogeneous texture than the one of sample B, indicating that on GaN the nucleation of κ phase should be enhanced.

HRTEM images were taken on both samples to investigate the nature of the boundaries between different areas; as shown in Figure 4 for sample B, the separation between the κ and β areas is well-defined. Similar results have been observed for sample A (Figure S4), and no relevant differences at this scale between the two samples have been found.

**Figure 4:**
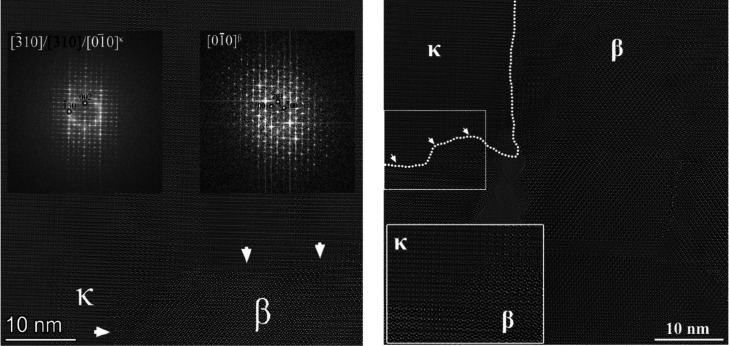
HRTEM images of sample B on GaN, showing the boundary between the κ and β areas with arrows and with enlarged cuts. Indexed FFTs show the orientation relationship between the two crystals (κ and β).

These observations suggest that κ-Ga_2_O_3_ and β-Ga_2_O_3_ grow at the same time: the vertical elongated area of grains and the zigzag grain boundaries at about 10 nm step support this interpretation, hinting to the fact that the growth window for these materials overlaps, and for the GaN template, this window is shifted at lower supersaturation with respect to sapphire.

### 3.2 Structural Analysis of the Interface Layer

In Figure 5, HRTEM images of the interface region for point 1 of both samples A and B are shown, in areas where pure κ-Ga_2_O_3_ grows in the bulk of the deposited layer.

**Figure 5:**
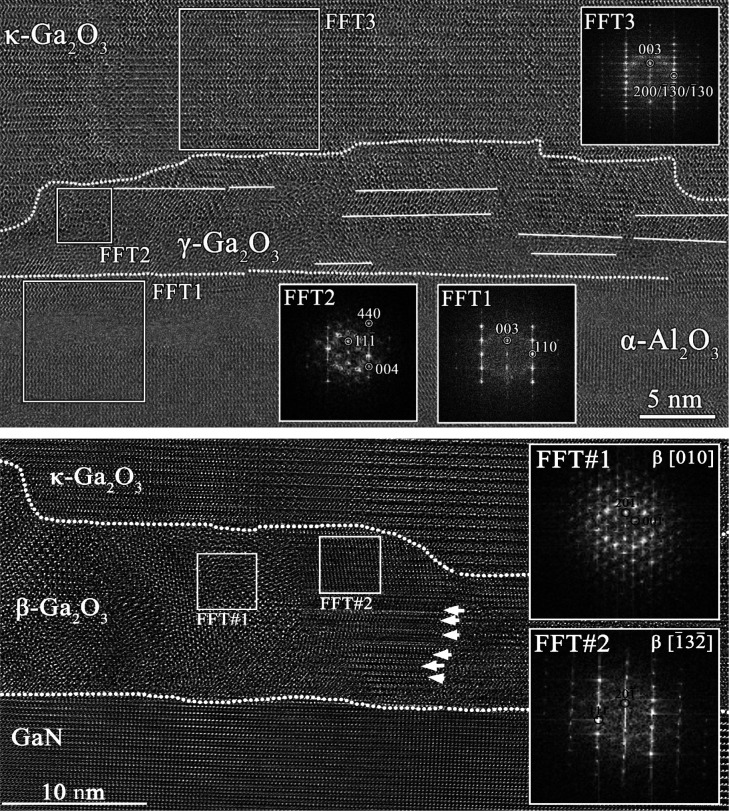
HRTEM images of interface for samples A (top) and B (bottom), with indexed FFTs showing the orientation of the identified phases. The white arrows highlight extended defects.

For sample A (grown on sapphire), a γ-phase Ga_2_O_3_ interlayer is found between the α-Al_2_O_3_ substrate and the κ-Ga_2_O_3_ film, with thickness below 10 nm and orientations (111) γ-Ga_2_O_3_||(001) α-Al_2_O_3_ and (112) γ-Ga_2_O_3_||(110) α-Al_2_O_3_. Twin boundaries (2-fold rotational or mirror plane) parallel to (111) γ are present, resulting in additional components in the FFT of the HRTEM image, providing the relationships (11–1) γ-Ga_2_O_3_||(001) α-Al_2_O_3_ and (224) γ-Ga_2_O_3_||(110) α-Al_2_O_3_.

In sample B (grown on GaN), the interface layer is identified as a textured β phase. Main orientations are in the [010] β projection: (20–1) β-Ga_2_O_3_||(001) GaN and (020) β-Ga_2_O_3_||(−120) GaN and in the [−13–2] β projection; (20–1) β- Ga_2_O_3_||(001) GaN and (020) β is almost parallel to (−210) GaN within 1°. Some features, highlighted through white arrows in Figure 5, can be associated with planar faults in β-Ga_2_O_3_ or Moiré patterns of two β-Ga_2_O_3_ lattices. It is noteworthy that no γ-Ga_2_O_3_ has been found in this case. Summarizing the results at point 1, close to the inlet, we can say that a β/γ interlayer is present at the interface with the substrate and that the κ phase eventually grows on top, independent from the presence of inclusions of the γ phase. Actually, most recent studies have recognized the structural analogy between the γ and β phases, except for the presence of some disorder (see also ref. 44 and the references therein). Because the only difference between samples A and B is the substrate, the presence of the γ phase can thus be associated with a different amount of strain due to the different mismatch, as discussed in Section 3.3.

To investigate this point further, the thinner sample C grown on sapphire was also analyzed, and its HRTEM image is shown in Figure 6 (top).

**Figure 6:**
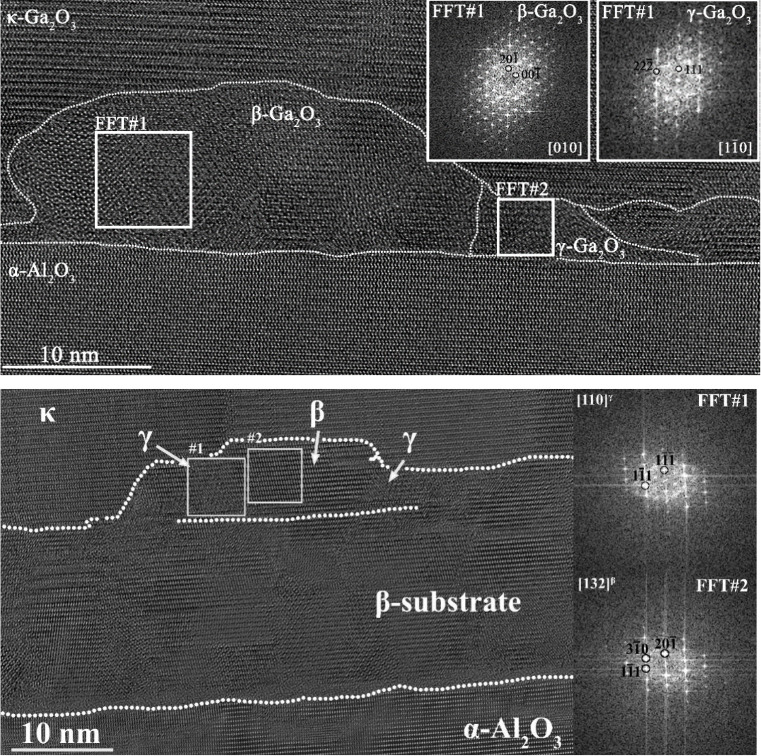
HRTEM images of the interface for sample C on sapphire (top) and sample D on β-Ga_2_O_3_ template (bottom), with indexed FFTs showing the orientation of the identified phases.

The interlayer appears morphologically different from the thicker sample A: its thickness is between 5 nm and 25 nm and much less uniform. Two different phases have been identified: mainly β with inclusions of γ. Where β-Ga_2_O_3_ is present, the thickness of the interlayer reaches 25 nm, while the layer is thinner where γ-Ga_2_O_3_ is dominant. . The orientational relationships are (111) γ-Al_2_O_3_||(20–1) β-Al_2_O_3_||(001) α-Al_2_O_3_ and (22–4) γ-Al_2_O_3_||(110) α-Al_2_O_3_ with other orientations of β also occurring.

It should be stressed that samples A and C were grown in the same experimental conditions (growth rate, temperature, and precursor flows), with the only parameter modified being the growth time (20 min instead of 120 min). Therefore, we interpret this observation of larger amounts of γ phase in the interlayer of sample A as the result of a phase conversion mechanism from β-Ga_2_O_3_, which has been reported so far just for thermally annealed or ion-implanted samples.^16,34–36,45^ An atomic-scale mechanism for this transformation due to the presence of interstitial defect complexes has been proposed,^46^ and other reports suggest that the metastable γ-Ga_2_O_3_ phase can be a preferred configuration over an highly defective β-Ga_2_O_3_ due to strain accumulation.^45,47^

To further support this interpretation of a β-to-γ-phase conversion of a highly defective β-Ga_2_O_3_ during the growth, sample D was also characterized by TEM imaging (Figure 6). This sample consists of a κ-Ga_2_O_3_ layer grown on top of a MBE (−201) β-Ga_2_O_3_ template on c-plane sapphire (large-area bright-field TEM image shown in Figure S5). Upon additional MOVPE deposition, the MBE interlayer remains pure β-Ga_2_O_3_. On top of that, the MOVPE-deposited material is not immediately starting as the κ-Ga_2_O_3_ film (which is dominating the bulk of sample D), but it is initially constituted by an additional β-Ga_2_O_3_ interlayer, in which however it is possible to highlight small inclusions of γ-Ga_2_O_3_ [Figure 6 (bottom)] , also confirmed by additional HRTEM investigations into different areas of the same sample (Figures S6 and S7) . We deem the observation of a different number of γ inclusions to be related to the different quality/defectivity between the MBE and MOVPE β interlayer ; however, this phenomenon will be the target of a future study, and it will not be further discussed in the framework of this work. Nonetheless, all our samples show the subsequent growth of κ-Ga_2_O_3_ irrespective of the presence of any inclusions of γ phase. Moreover, our TEM and GPA data confirm that the κ-overgrowth is unaffected by the possible β-to-γ transition (see the discussion in the next section).

### 3.3 Strain Analysis

In order to provide additional information on the strain in the different layers of our samples, we performed GPA on HRTEM images, as described in Section 2.4.

In Figure 7, we show the TEM and GPA images of sample A at position 3. Two different areas were studied by GPA, one passing through two large and thick domains of the β-interlayer (box I in Figure 7) and another including a very thin β-interlayer below the κ phase (box II in Figure 7).

**Figure 7:**
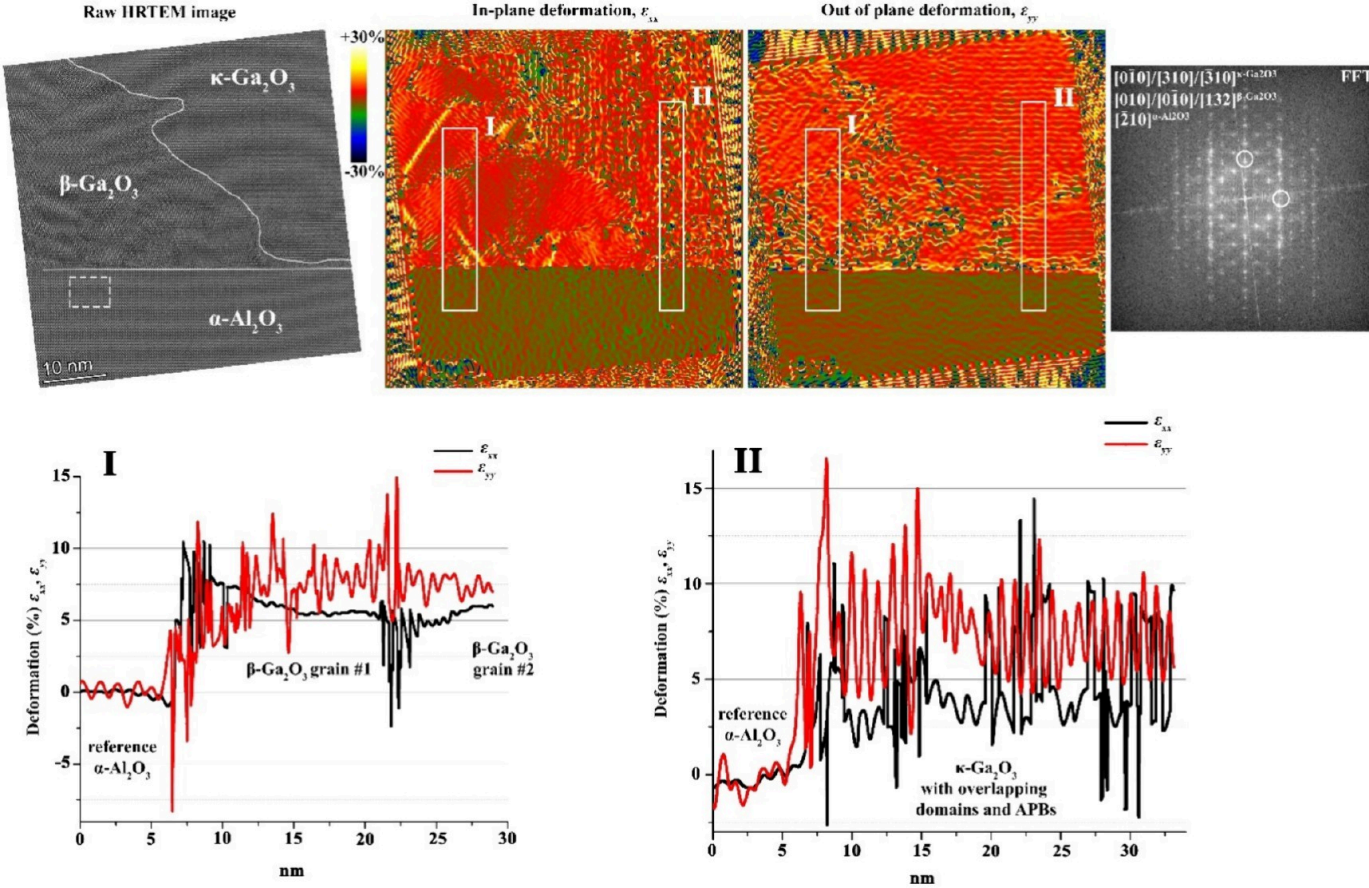
HRTEM image and deformation/strain maps calculated by GPA from sample A at position 3. The integrated deformation/strain profiles shown in the lower panels are taken from two different areas, highlighted as boxes I and II, one passing through two large and thick domains of the β-interlayer (left) and one with a very thin β-interlayer below the κ phase (right). Errors are within the ±0.02 range. The region taken as a reference for GPA is highlighted by the dashed rectangle on the HRTEM image. In its FFT plot, reported in the top-right panel, the two sets of diffraction spots selected for the analysis are marked by white circles.

We highlight that in this case the strain calculated by GPA is not the actual deformation of the investigated crystals but rather the apparent deformation of the atomic periodicity related to a fixed reference area arbitrarily chosen in the HRTEM image (in our case the substrate). Therefore, in order to quantify the actual strain in the epilayer, it is first necessary to calculate the strain value expected from the GPA for fully relaxed Ga_2_O_3_ crystals. Indeed, dealing with diffraction phenomena, it is not consistent to compare directly the bulk lattice constants of the materials but rather the distances between defined Miller planes (along the direction of the TEM lamella), therefore accounting also for the angles between the crystal axes. By inspecting the FFT of different regions of the HRTEM image, we identified three FFT components, one for each the Al_2_O_3_, β-Ga_2_O_3_, and κ-Ga_2_O_3_ crystals, which are close in reciprocal space (highlighted by white circles in Figure 7), in order to compare simultaneously their lattice periodicities. Because β does not show any low-index spot along the in-plane directions, we performed the calculation using the closest (to the axis) FFT component. In this case, an angle distortion on the phase map is present, and the GPA is calculated with the in-plane projection, in the reciprocal space, of this periodicity. It was necessary to choose this reflection because, from other zone axis of β, all of the grains are overlapping. Hence, for each of the chosen reference FFT spots, we calculated the corresponding distance between the reflection planes, which we report in Table S2.

In the case of c-sapphire substrate, for fully relaxed [010]-oriented β grains, we expect a ∼3%/8% apparent in-plane/out-of-plane tensile strain, and for [132]-oriented β grains, the apparent in-plane tensile strain should be ∼8% referred/compared to the α-Al_2_O_3_ substrate. The κ relaxed lattice should appear as expanded by ∼5–6% in the in-plane direction and ∼7% in the out-of-plane direction.

We can now inspect the GPA data along the TEM lamella that we have recorded for our Ga_2_O_3_ films. In Figure 7, from sample A, the experimental in-plane β deformation value (∼7%) is between the calculated values for [010]- and [132]-oriented β grains; however, the experimental value is strongly dependent on the misorientation and overlapping of the β grains. For sample D, as shown in Figure 8, the in-plane β deformation value is ∼7–8%, which is close to the value for [132]-β. Similarly, the film of κ-Ga_2_O_3_ appears as fully relaxed in Figures 7 and 8. In the case of the in-plane deformation values of κ in Figure 7, some residual strain could be present; however, the examined area of κ was affected by domain and antiphase boundaries.

**Figure 8:**
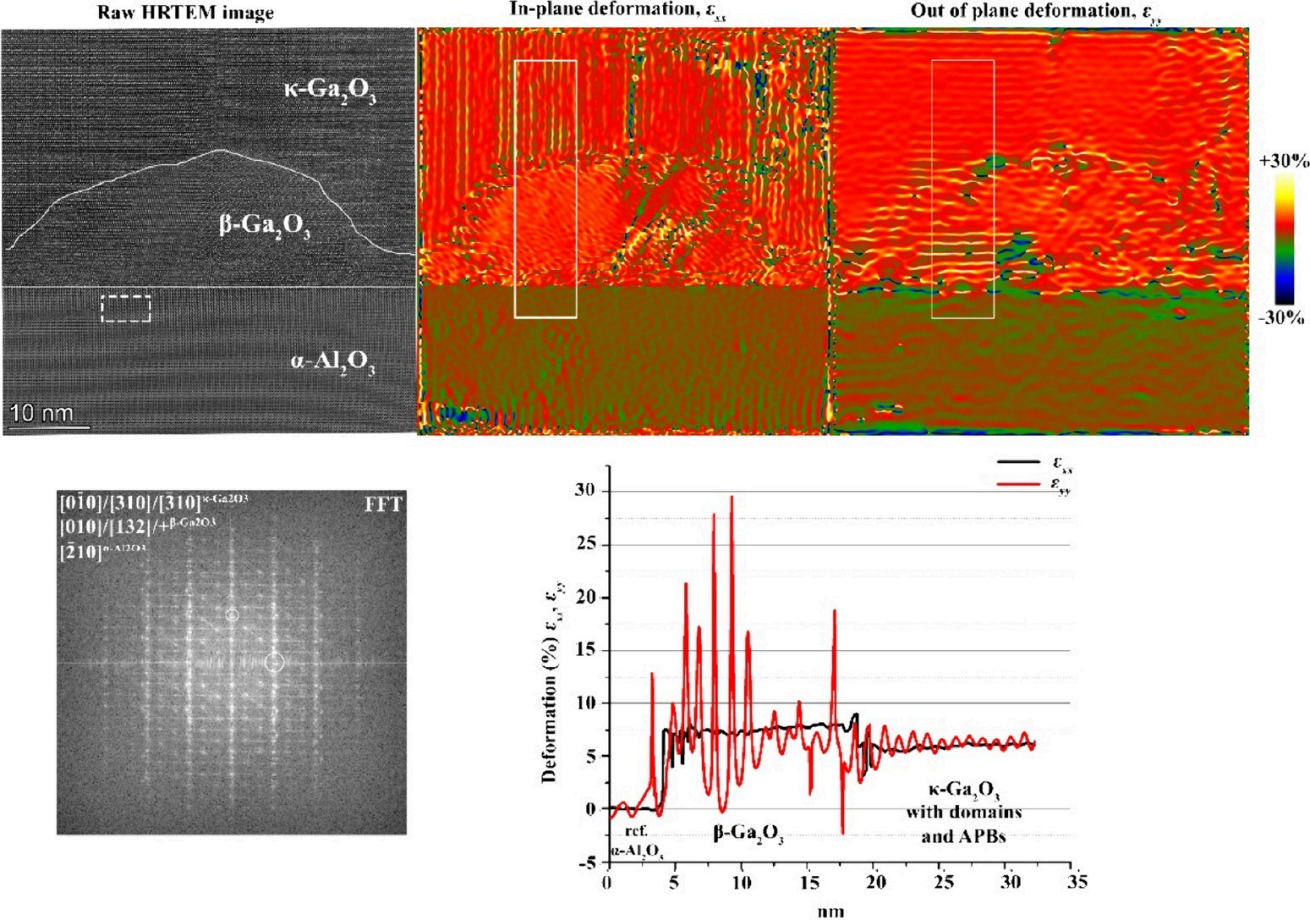
HRTEM image and ε_*xx*_ and ε_*yy*_ deformation/strain maps calculated by GPA from sample D at position 1. The integrated deformation/strain profile is calculated passing through the β-interlayer, highlighted by a white box. Errors are within the ±0.02 range. The region taken as a reference for GPA is highlighted by the dashed rectangle on the HRTEM image. The two sets of diffraction spots selected for the analysis are marked by white circles on the FFT plot.

In the case of the GaN substrate (sample B) for fully relaxed β, we expect in-plane and out-of-plane strain values of ε_*xx*_ ∼ −11% for [010]-β, ε_*xx*_ ∼ −6 to –7% for [132]-β, and ε_*yy*_ ∼ −10%. The apparent strain values that we calculated for a fully relaxed κ film should be ε_*xx*_ ∼ −8 to –9% and ε_*yy*_ ∼ −10 %. From Figure 9, we see that the experimental values that we obtain from the GPA for κ are −8% and −12%, respectively, which are close to the calculated values. For β-Ga_2_O_3_, the experimental out-of-plane value of ∼−10% is similar to the expected one for a fully relaxed crystal. In the case of the in-plane strain, the presence and overlap of very small (in some cases, a few nanometers large) β grains make the GPA evaluation process less straightforward. Indeed, from the strain map reported in Figure 9, we observe the presence of a spot with an extremely high “tensile” value (∼20%), which may be the result of the possible incorporation of [-2 0 1] reflections (with a *d* spacing of 3.668 Å) due to overlapping β grains. Nonetheless, the average of the overall apparent in-plane strain plot attributed to the β phase is ε_*xx*_ = −0.065 ± 0.023, which is close to the calculated value for relaxed [132]-β.

**Figure 9:**
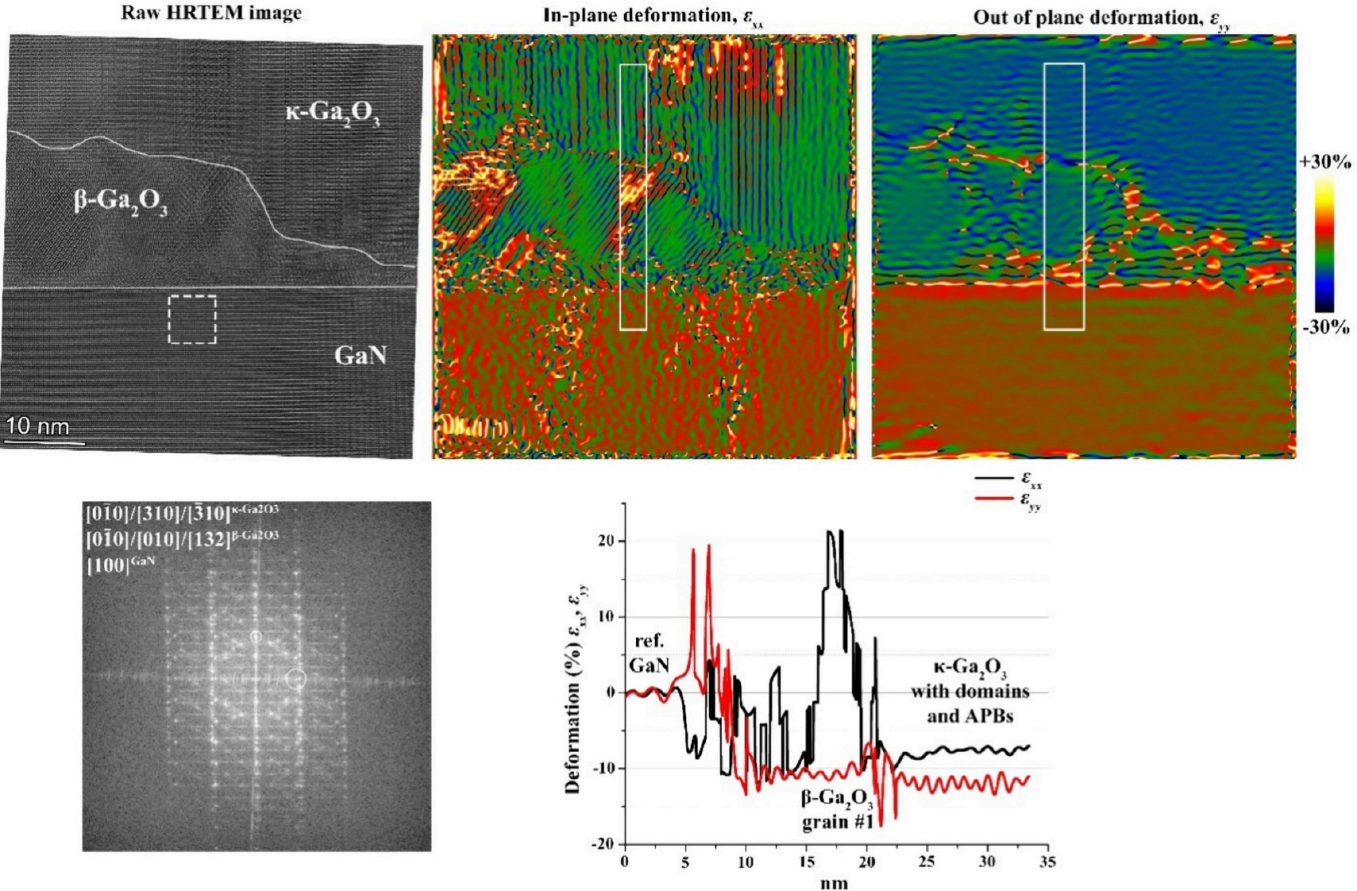
TEM and GPA images of sample B at position 1. HRTEM image with ε_*xx*_ and ε_*yy*_ deformation/strain maps calculated by GPA from sample B at position 1. The integrated deformation/strain profile is calculated passing through the β-interlayer, highlighted by a white elongated box. Errors are within the ±0.02 range. The region taken as a reference for GPA is highlighted by the dashed rectangle on the HRTEM image. The two sets of diffraction spots selected for the analysis are marked by white circles on the FFT plot.

## 4 Nucleation Model

To provide a rationale for the observed competition between the different Ga_2_O_3_ polymorphs during the epitaxial growth, we elaborated a thermodynamic nucleation model, accounting for both the effect of precursor gas supersaturation and the misfit strain imposed by the substrate. Here we provide the key concepts behind the formulation of the model, the definition of its parameters, and the main results. Additional information is available in the Supporting Information.

### 4.1 Model Definition

From classical nucleation theory, the change in free energy Δ*G* when *N* Ga_2_O_3_ formula units (f.u.) in the gas phase crystallize into a 2D or 3D island onto the epitaxial substrate can be written, respectively, as

1 $${\Delta G}_{2D}\left(N\right)=-\left(\Delta \mu -{\Delta \mu }_{2D}^{\varepsilon }-\Delta \gamma \nu /h\right)N+\Lambda \sqrt{N}$$

2 $${\Delta G}_{3D}\left(N\right)=-\left(\Delta \mu -{\Delta \mu }_{3D}^{\varepsilon }\right)N+\nu^{2/3}\Gamma N^{2/3}$$

Δμ = μ_gas_ – μ_solid_^bulk^ is the supersaturation (per f.u.) of the gas with respect to the solid in its relaxed bulk phase, and Δμ_2D_^ε^ and Δμ_3D_^ε^ are the elastic energy densities per f.u. in the 2D or 3D island configurations. Δγ = γ_epi_ + γ_int_ – γ_sub_ accounts for the energy change per unit area when exposing the top surface of a 2D epilayer (with surface energy density γ_epi_) while covering the substrate (γ_sub_) and forming the interface (γ_int_). *h* is the height of the 2D layer and ν is the volume per f.u., so that the covered area is ∝*Nν*/*h*. In principle, the supersaturation and the other parameters of the model also depend on the growth pressure and temperature. Nonetheless, we here take their values from the first-principles calculations based on the density functional theory (DFT) at 0 K, reported in refs. 29 and 38 (Table S3), because the ranking of the free energies calculated for the different Ga_2_O_3_ polymorphs has been found to be weakly sensitive to the macroscopic growth conditions.^39^ Λ is the effective step-edge energy density, accounting for the perimeter cost of a finite-sized 2D island. Given the lack of experimental evidence about such islands and the prohibitive cost for inspecting realistic step-edge structures, we here assume $\Lambda =\lambda C_{p}\sqrt{\nu /h}$ , with λ being a free-parameter, bounded in the range of 50–100 meV/Å, common to several material classes,^48–50^ and *C*_p_ = 3.7 is a geometric correction approximately valid for the prototypical island (see the Supporting Information). Finally, Γ is the effective surface energy density (per f.u.) for a 3D island accounting for the cost of exposing facets. It can be calculated as an average of surface energy densities γ_*i*_ of all exposed facets, each weighted by a geometrical factor *c*_*i*_ accounting for its relative area, and including the energy variation due to expansion of the island interface and covering the substrate and, i.e., Γ = ∑_*i*_ *c*_*i*_γ_*i*_ + *c*_bot_(γ_int_ – γ_sub_).

In order to evaluate Γ and Δμ_3D_^ε^, the island shape must be defined. Here we consider 3D islands only of the β and κ phases. We modeled the former as truncated cones mimicking the shape and the aspect ratio of islands experimentally observed by AFM and scanning electron microscopy measurements, shown in Figure S8, and taking an average value of typical low-index facets for the lateral surface (see the Supporting Information). For the latter, we instead consider a hexagonal prismatic shape with 6-fold rotational domains, as already reported in the literature,^51,52^ with {130} sidewalls for which we calculated the surface energy density γ_130_ by DFT (Figure S9). For both 3D-β and 3D-κ geometries, we then evaluated Δμ_3D_^ε^ by solving the mechanical equilibrium problem via finite-element method (FEM) calculations using the COMSOLⓇ Multiphysics software, setting the appropriate anisotropic elastic constants for both the Ga_2_O_3_ island and c-sapphire substrate, as obtained from DFT simulations of the oriented bulk. Additional information about the details of the FEM calculations is reported in the Supporting Information.

Equations 1 and 2 account for the balance between the gain in energy when forming the solid (in volume) and the cost associated with the formation of a finite-sized island, namely, the cost of its perimeter in 2D or of its surface in 3D. Then, the nucleation work Δ*G* ^crit^ for each type of island can be identified as the maximum of the Δ*G*(*N*) curve, which parametrically depends on the gas supersaturation. By simple differentiation, we obtain

3 $${\Delta G}_{2D}^{\mathrm{crit}}\left(\Delta \mu \right)=\frac{\Lambda^{2}}{4\left(\Delta \mu -{\Delta \mu }_{3D}^{\varepsilon }-\Delta \gamma \nu /h\right)}$$

4 $${\Delta G}_{3D}^{\mathrm{crit}}\left(\Delta \mu \right)=\frac{{4\nu }^{2}\Gamma^{3}}{27{\left(\Delta \mu -{\Delta \mu }_{3D}^{\varepsilon }\right)}^{2}}$$

The corresponding formulas for the size of the critical nuclei *N* ^crit^ can be obtained straightforwardly as *N*_2D_^crit^ = Δ*G*_2D_^crit^/(Δμ – Δμ_3D_^ε^ – Δγν/*h*) and *N*_3D_^crit^ = 2Δ*G*_3D_^crit^/(Δμ – Δμ_3D_^ε^).

Finally, the nucleation rate of any islands can be analytically expressed as

5 $$J\left(\Delta \mu \right)=\frac{\sqrt{\frac{{\Delta G}^{\mathrm{crit}}}{k_{B}T}}}{N^{\mathrm{crit}}}e^{-{\Delta G}^{\mathrm{crit}}/k_{B}T}$$

It must be remarked that all quantities so far reported are distinct for each Ga_2_O_3_ phase, either for 2D or 3D islands. In order to make consistent comparisons, a common reference must be set: the most natural choice is the phase having the lowest bulk chemical potential, namely, β-Ga_2_O_3_. In the following, we then refer all values of supersaturation Δμ to such bulk chemical potential.

### 4.2 Gas Supersaturation and Alignment with DFT Data

Even though our theoretical model depends on the gas supersaturation, to use it for a quantitative analysis of the different experimental cases, we first need to evaluate its value at the actual growth conditions and then align it with the energy scale of the numerical chemical potentials for the different bulk phases. This cannot be easily achieved because (i) we do not directly know the absolute values of supersaturation, since only the nominal partial pressure of the precursor at the inlet is measured, and (ii) DFT calculations do not provide an absolute value of the chemical potential of the bulk to be compared with that of the precursors in the gas phase.

As a starting point to determine the gas supersaturation, we then treat the mixture of precursor gases, i.e., TMGa and H_2_O, as an ideal gas; therefore, we consider ${\Delta \mu }^{\exp t}=k_{B}T\;\ln\left(\frac{p_{\mathrm{growth}}}{p_{\mathrm{eq}}}\right)$ . To evaluate the ratio of partial pressures at any grown condition, we follow an empirical approach, based on taking the TMGa as a limiting agent for the epitaxial growth and assuming a linear relationship between both the nominal inlet flux (*F*_TMG_) and the growth rate (GR) of the Ga_2_O_3_ crystal, which are those reported in ref. 15 and measured in the same growth chamber and under the same growth conditions of the layers considered in this paper. Heuristically, we assume as equilibrium condition, i.e., Δμ_expt_ ≈ 0, the experimental case corresponding to the lowest nominal flux at the outlet of the sample (i.e., *F*_TMG_^1/18^ in ref. 15), where β-Ga_2_O_3_ 3D islands are observed to grow at very low rate. In this way, it becomes possible to evaluate the ratio $\frac{p_{\mathrm{growth}}}{p_{\mathrm{eq}}}$ , through the combined effect of the ratio of the fluxes at the inlet, accounting for the different nominal amounts of TMGa and the ratio of the growth rates at the different *x* positions of the sample, for a fixed flux of precursors, as $\frac{F_{\mathrm{TMG}}^{\mathrm{growth}}}{F_{\mathrm{TMG}}^{1/18}}\frac{GR\left(x\right)}{GR\left(\mathrm{outlet}\right)}$ . This range of experimental supersaturation values is sketched in Figure 10a, using the data from ref. 15 (see additional details in the Supporting Information, along with Table S4), including samples grown at both 610 and 650 °C.

**Figure 10:**
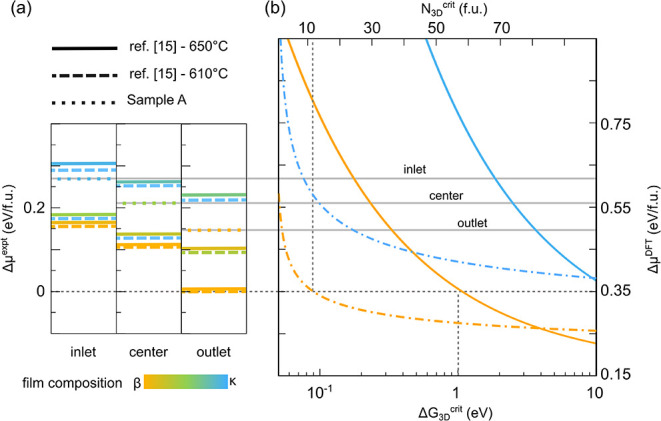
Comparison of the supersaturation as estimated from experiments (a) and the scale of numerical chemical potentials (b) through the evaluation of the nucleation barrier (solid line) and the size (dot-dashed line) of the critical nuclei for 3D-β islands (orange lines). For reference, the curves calculated for 3D-κ islands are alsoshown. The supersaturation values estimated in the experiments for sample A are highlighted with horizontal gray lines. In panel b, the dotted gray line marks the nucleation thresholds for Δ*G* ^crit^and *N* ^crit^ and the corresponding reference chemical potential.

Now, we need to align such values Δμ^expt^ with the numerical Δμ^DFT^ scale for the chemical potential of the solid phase, having the thermodynamically stable β bulk phase as the reference. Because our Δμ^expt^ ≈ 0 condition was chosen as the onset for the nucleation of small 3D-β islands, we align it with the numerical Δμ^DFT^ value at which our model predicts reasonably small nucleation barriers (∼1 eV) and critical nucleus size of just a few Ga_2_O_3_ formula units for the 3D-β islands only, as indicated in Figure 10b. For the sake of convenience, in Figure 10b as well as in all following plots, we include as guidelines the values of gas supersaturation at the inlet, center, and outlet for sample A only. The other cases for 610 and 650 °C follow the same growth regimes, due to the very similar supersaturation values (Figure 10a).

### 4.3 Model Predictions of Early Growth Stages on the (0001) Al_2_O_3_ Substrate

In Figure 11, we report our results of Δ*G* ^crit^ and *J* for the nucleation of isolated 2D and 3D islands of β- and κ-Ga_2_O_3_ on the sapphire substrate, while the corresponding plots for *N* ^crit^ are shown in Figure S11. We included in our model also 2D islands of the α phase, in order to possibly account for the formation of a thin wetting layer of this polymorph, as sometimes reported in the literature.^18^

**Figure 11:**
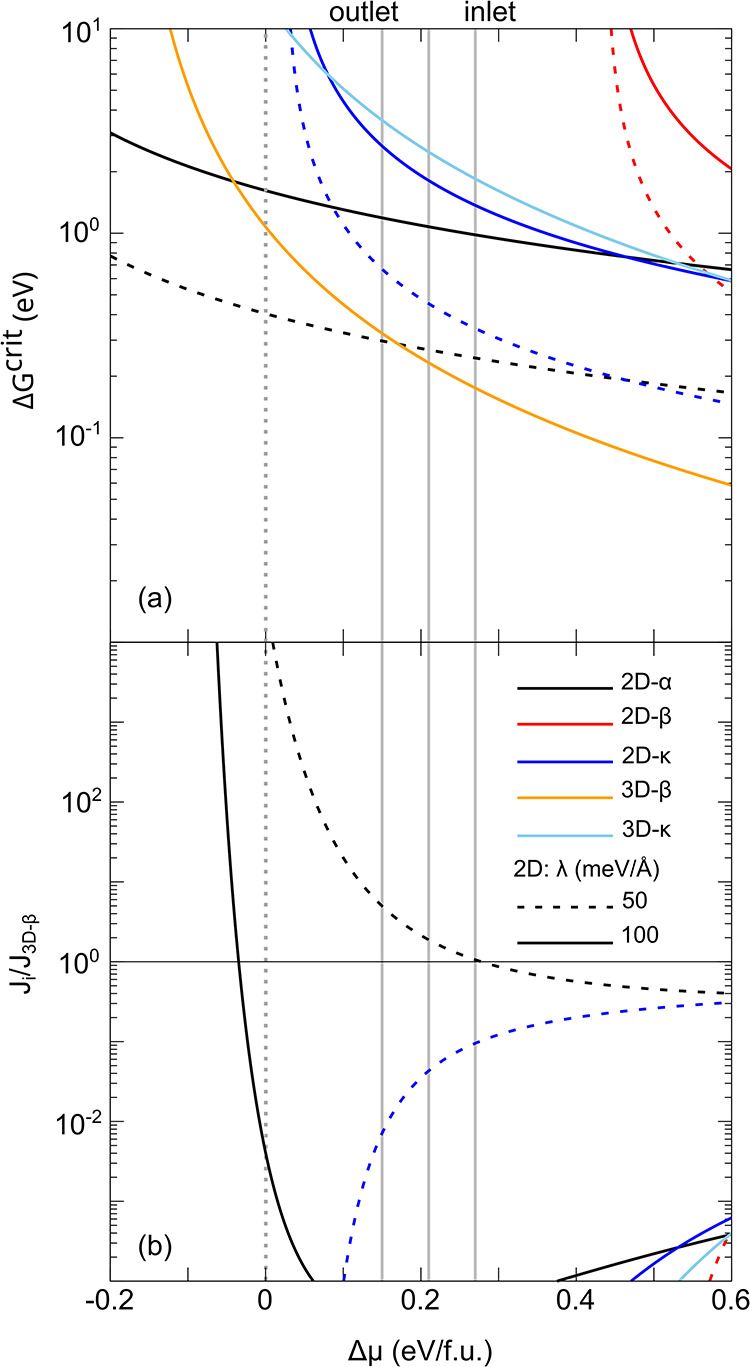
Nucleation barriers (a) and nucleation rates (b) calculated for 2D or 3D islands of different Ga_2_O_3_ polymorphs on the Al_2_O_3_ substrate, with different values of the step-edge energy λ. In panel b, the data shows the ratio of the calculated rates vs that of the 3D-β island. The vertical gray solid lines mark the estimated supersaturation for different points on the samples.

Within the supersaturation window estimated for sample A (delimited by vertical gray lines), the free energy barrier calculated for the nucleation of 3D-β islands (Figure 11a) is lower than that of any other phase. Correspondingly, the nucleation rates of the phases/island shapes are much lower than those of the 3D-β islands taken as reference, as can be seen in Figure 11b, where the curves representing their ratios are much lower than 1. This advantage is driven by the large elastic energy reduction allowed by the formation of 3D islands and the lowest free surface energy of the (−201) β-Ga_2_O_3_ surface. This result is particularly evident if we assume that the 2D island step-edge energy λ (here taken the same for all 2D islands, for simplicity) is about 100 meV/Å rather than 50 meV/Å, i.e., our maximum value (solid vs dashed lines in Figure 11). The case of 2D-α islands is an interesting exception. From Figure 11b, it is indeed possible to observe that, for sufficiently low Δμ, the nucleation rate of this phase (2D) becomes larger than the one of 3D-β islands. This is possible thanks to its negligible interface energy with the underlying Al_2_O_3_ substrate (Table S3) and is further enhanced when considering a reduced step-edge energy (closer to 50 meV/Å). While none of the experimental cases under scrutiny meets such low Δμ conditions, it is still possible that different experiments could fall within such a region. This might be the case of refs. 16 and 18, where a thin α interlayer was first reported to growth, before reverting to the 3D-β islands growth, as discussed in the Supporting Information, Section 5 (see also Figure S10).

### 4.4 Numerical Study of κ-Ga_2_O_3_ nucleation on β-Ga_2_O_3_ interlayer

In order to provide some insights into the later stages of the growth, we refer to the sample structure observed by TEM in Figures 5–9. It is indeed clear that the thick films of both β and κ phases growing in the latest stages develop on top of a (few nanometers) thin interlayer of the β phase, expected to result from the full coalescence of the 3D-β islands previously formed on the bare sapphire substrate. As coalescence reduces the morphological degrees of freedom for the strain release, such a β interlayer will present a residual misfit with respect to underlying sapphire, possibly larger than the average misfit in the starting 3D islands. Therefore, we consider the β interlayer as the actual substrate, on top of which the β and κ islands nucleate to continue the growth and we compute their nucleation work Δ*G* ^crit^ and rates *J*. It is expected that only 2D islands matter at this stage because forming again 3D-β islands on top of the coalesced film is quite unrealistic. Moreover, as will be shown in a moment, 3D-κ islands require a much higher nucleation work because of the larger surface/volume ratio compared with the 2D island and result in an overall larger surface energy compared with the β phase. The resulting nucleation curves are reported in Figure 12.

**Figure 12:**
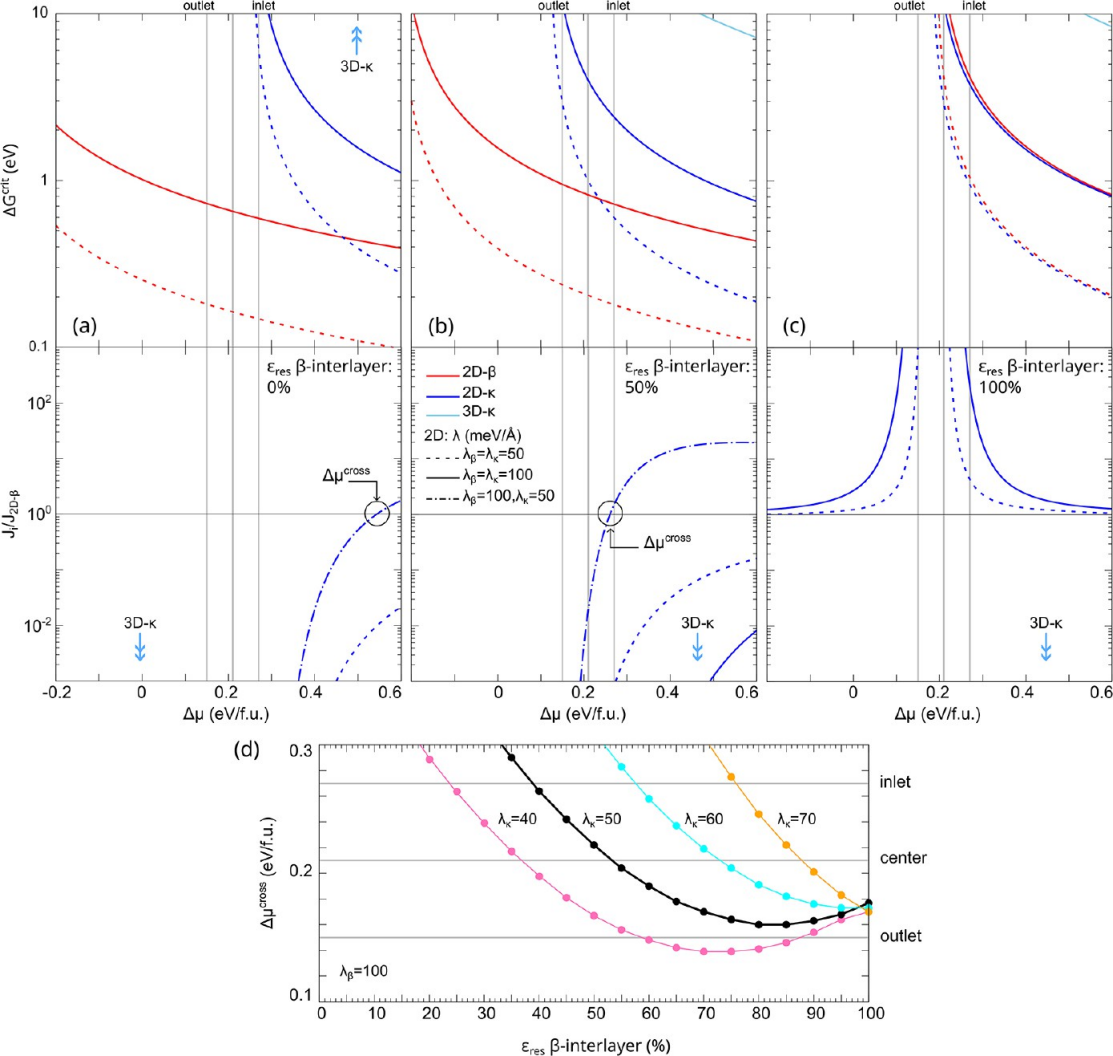
Comparison between the nucleation barrier (top panels) and ratio of the nucleation rates vs 2D-β islands (bottom panels) of 2D-β and 2D/3D-κ Ga_2_O_3_ islands on the β-Ga_2_O_3_ template considered with different strain states (ε^res^): fully relaxed (a) and at 50% residual strain vs c-sapphire lattice (b) and fully strained vs c-sapphire lattice (c). The light-blue arrows indicate that the plots for 3D-κ islands are outside the range of the graphs. Panel d shows the supersaturation value (Δμ^cross^) where *J*_2D‑β_ is a function of ε^res^ for different combinations of the step-edge energy of the two islands. Different values of the edge-energy λ are included as well. The vertical gray lines highlight the supersaturation values estimated from the experimental growth conditions.

For a fully relaxed β interlayer (Figure 12a), the nucleation of 2D-β islands is by far the most favored at all supersaturation regimes, as expected for homoepitaxy. On the contrary, if the interlayer remains fully strained (Figure 12c), the nucleation barriers of 2D-κ islands are practically the same as those calculated for 2D-β and the ratio of the nucleation rates is always ≫1 for our experimental supersaturation values.

When considering a β interlayer with partial strain relaxation some interesting effects can be observed, as exemplified for the prototypical case of 50% residual misfit reported in Figure 13b. Assuming the same value of the step-edge energy for both β and κ 2D islands, the nucleation of the β phase is predicted to be by far the most favorable. However, more realistically, a different λ should be expected for the islands of the two phases, reasonably limited to less than a factor 2 to remain in the typical range of step-edge energies. If we admit that the λ for 2D-β islands is sufficiently larger than that of the 2D-κ ones, it is possible to obtain that their Δ*G* ^crit^ curves intersect, thus inverting their nucleation probabilities *J* and hence making growth of the κ phase more convenient at higher supersaturation values. This is exemplified by the dot-dashed line curve in the plot of *J* in Figure 13b, where we attribute the highest λ value of 100 meV/Å to the 2D-β islands and the lowest, i.e., 50 meV/Å, to the 2D-κ islands. In such a case, we obtain that for the high supersaturation values in the inlet region, the nucleation of κ-phase islands is slightly favored against the β ones, while the latter becomes prevalent when moving toward the outlet at low supersaturation. Simultaneous nucleation and eventually coexistence between the two phases can then be expected at intermediate conditions as in the wafer center.

**Figure 13:**
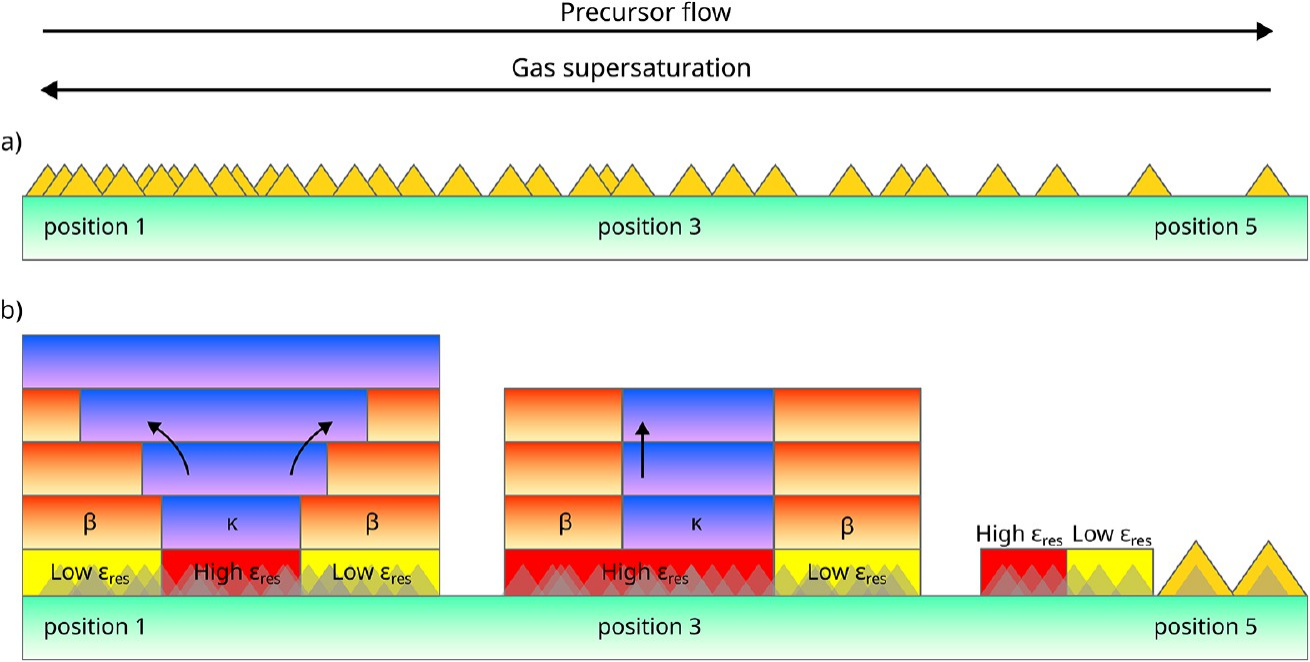
Sketches of the evolution of MOVPE growth of the Ga_2_O_3_ polymorphs. Panel a shows the different nucleation mechanisms, depending on the growth conditions and the strain state of the interlayer. Panel b reports a scheme of the evolution of the competing β and κ grains. In both panels, the relative positions on the experimental samples are indicated.

In order to provide a more systematic evidence of the relationship between strain relaxation and supersaturation in controlling the β/κ phase nucleation competition, in Figure 12d, we evaluate the critical supersaturation Δμ^cross^ at which the nucleation rates of the two phases are the same (i.e., *J*_2D‑κ_/*J*_2D‑β_ = 1), as a function of the residual strain in the β-Ga_2_O_3_ template. Different values of the step-edge energy of the 2D islands of the two phases are also considered. In the first case, where the step-edge energy ratio of the β/κ phases is the highest in the range that we have considered so far, i.e., λ_β_/λ_κ_ ∼ 2, it is sufficient to consider a residual strain slightly above 50% to favor the nucleation of 2D-κ islands at the center region of the sample (point 3). By a reduction of the λ_β_/λ_κ_ ratio, the free energy advantage of the islands of the metastable Ga_2_O_3_ phase quickly reduces, while Δμ^cross^ increases, even exceeding the maximum supersaturation in our experiments, thus making the β islands the only possible option. Vice versa, if admitting a step-energy for the 2D-κ value lower than our lower bound of 50 meV/Å^2^, the formation of the κ islands would be dominant even at supersaturations lower than those of the outlet, in contrast with our experimental findings.

As a general consideration, our model highlights that the nucleation of the κ-Ga_2_O_3_ phase stems from a delicate interplay between the elastic energy and surface/interface energy differences between the different phases rather than just from a different strain contribution, as recently proposed in the literature.^44^

Finally, we have also estimated the nucleation curves in the case of a β-Ga_2_O_3_ substrate with variable residual strain, this time on a (0001) GaN surface. In Figures S12–S14, we provide a rationale for the calculation of the lattice misfit for commensurate interfaces between β and κ phases with the sapphire (0001) substrate and with a GaN (0001) substrate, within the hypothesis that a network of O atoms is created also on the GaN surface, when the β-Ga_2_O_3_ film is deposited on top. We see that while the misfit is always compressive for the sapphire, it becomes tensile in the case of GaN. This is confirmed by the GPA data in Figure 9. While we cannot investigate the nucleation of the β-islands directly on the GaN substrate, because interface energies for such a case are not available, we estimated the strain in the interlayer and the interface energy between the κ and β layers. We highlight that we have evaluated the elastic energy density of 2D islands of β and κ phases under two hypothetical situations, i.e., (i) keeping the common network of O atoms at the interface, which is expected to minimize the interface energy, or (ii) modifying the epitaxial relationship in order to minimize the misfit strain, at the expense of increasing the interface energy. Further discussion about these calculations can be found in the Supporting Information. As shown in Figure S15a–c, the results of the most favorable scenario are similar to the ones we found for the case of the Al_2_O_3_ substrate, where the nucleation of 2D-κ islands gets triggered for higher (∼50%) residual strain in the β film. In comparison with the case of the Al_2_O_3_ substrate, the elastic energy density in the κ-Ga_2_O_3_ film grown on top of the β film with an underlying GaN substrate is notably smaller (Figure S15d); however, such a free energy gain is only partly compensated for by an increased interface energy between the two films. Such a balance is indeed a delicate quantity to calculate; nonetheless, our qualitative model in the case of GaN is able to capture the strong competition between the nucleation of the β or κ phase.

## 5 Interpretation and Discussion of the Progression of Stages of the Epitaxial Growth

In Figure 13a, we show a sketch of our interpretation of the epitaxial growth of Ga_2_O_3_ by MOVPE with respect to the supersaturation and growth time on sapphire and GaN. The different phase compositions of the film as a function of the position on the sample can be related to the interplay between the deposition time, supersaturation of the gases, and strain of the growth template at any stage.

As a first step, on both the sapphire and GaN substrates, the (small) 3D-β islands are likely to appear first, everywhere on the sample, although with different density. At those experimental conditions, i.e., a combination of short deposition times and/or low supersaturation, e.g., found at the outlet of sample A or with the lowest nominal flux of the precursors in ref. 15, the growth of the Ga_2_O_3_ film is terminated even before the coalescence of the 3D-β islands.

Next, the evolution of the strain with island extension to more domains and up to the coalescence of the islands is a crucial matter. On the one hand, it is very probable that the transformation from isolated 3D islands to a coalesced film leads to an increase of the elastic energy density, driven by the loss of the elastic energy release provided by the free (lateral) surfaces. On the other hand, it is likely that the domain boundaries and grain boundaries between different coalesced islands may provide some strain relaxation. TEM images (Figures 5–9) suggest that the crystal quality of the first few nanometers of the coalesced β-Ga_2_O_3_ interface layer is rather low, because a textured thin film is observed. This would suggest a nonuniform strain state close to the substrate, for the whole interface of the sample, given the proper growth conditions, namely, enough time and/or supersaturation. Indeed, from our GPA (Figures 7–9), we see that the thin β interlayer shows large oscillations of the lattice parameter along the growth front, but we really cannot say whether they correspond just to different crystallographic directions or to different strain values. Therefore, in the next step of the Ga_2_O_3_ film growth, the interlayer of β phase acts as a growth template, with the local relaxation of the lattice parameters playing the role of an additional parameter in the nucleation of β and κ phases, competing with supersaturation.

In those regions where the interlayer released most of the elastic energy through the formation of various structural defects, the continuation of the 2D growth of the β phase is expected by our predictions (Figure 12a), especially at the lowest supersaturation values. This is experimentally confirmed by the prevalent β polymorph found at position 5 (closer to the outlet) in the samples with the lowest nominal gas fluxes.

At those regions with higher residual strain, the growth hierarchy is strongly driven by supersaturation. At position 1 (high supersaturation, close to the inlet), we believe that, after the coalescence of 3D-β islands into a film, the nucleation of the 2D-κ island is possible and most convenient, as suggested by our calculations, at least in the portions of the β interlayer where the residual strain is around 40% or larger (Figure 12b,c). These regions most likely correspond to the very inhomogeneous, thin interlayer observed in the cross-section TEM images, formed by a large number of small grains. Possibly, high-strain regions may promote the formation of grains of γ-Ga_2_O_3_, but such a phase transformation is presumed to take place on a slower time scale, as suggested by the comparison of samples A and C (see Section 3.2) so that the 2D-κ island should nucleate on the β-coalesced layers. Anyway, given the structural affinity of γ- and β-Ga_2_O_3_, it is also likely that κ layers could even develop on γ regions. Even if some γ grains could form competitively with β ones, their structures are quite similar, as recognized from literature studies,^34,53^ possibly due to the common oxygen sublattice. Hence, a similar energy balance can be expected with respect to the nucleation of 2D-κ islands, which would eventually form and extend laterally on both β and γ regions.

Starting from these few critical nuclei, the lateral kinetic growth of the 2D-κ phase should take off at a rate larger than the β phase (Figure 13b), possibly covering, layer-after-layer, the β-Ga_2_O_3_ film, which might be described as a large 3D island. For example, in the TEM–GPA panels of Figure 8, we see that when a single, relaxed grain of the β phase is present, the lateral overgrowth by the κ phase is observed at larger thicknesses of the β domain, due to a minor imbalance in the growth rate. The same effect is suggested by panel a of Figure 9 in ref. 16, where the cross section between the β interlayer and the κ phase on top suggests that the multipinnacle-shaped profile of the β interlayer is produced by the progressive, lateral overgrowth by the κ phase nucleated at suitable strain conditions in between the pinnacles.

Finally, closer to the center of the sample (position 3), at intermediate supersaturation conditions, both the predicted nucleation rates (Figure 12b) and the expected lateral overgrowth velocity show a tight competition between the two phases, producing the columnar growth shown in Figure 3, partially switching from one phase to the other as the growth proceeds.

Given the similarities between the experimental and theoretical results that we found for the Al_2_O_3_ and GaN substrates, we deem the growth process to be qualitatively similar in the two cases. However, the samples grown over GaN show an enhanced nucleation of κ-Ga_2_O_3_ at the intermediate position (point 3). Indeed, our calculations evidenced that on GaN the absence of an interfacial network of O atoms between the β interlayer and the κ-phase epilayer provides an additional degree of freedom for the structure of the interface, enabling a pathway for plastic relaxation, at the expense of larger interface energies. This phenomenon depends on complex local strain relaxation mechanisms and affects the κ vs β competition, further favoring the growth of κ-Ga_2_O_3_ grains. Finally, it is worth stressing that interface energy and strain contributions are strongly influenced by the substrate symmetry.^54–56^

## 6 Conclusions

In this paper, we have investigated in detail the morphology and texture of the early stages of the MOVPE growth of Ga_2_O_3_ on (0001) Al_2_O_3_ and GaN substrates and compared these results with the predictions of a theoretical nucleation model based on first-principles parameters. Our study shows that the observed competition between β and κ phases is driven by both thermodynamics, i.e., different nucleation rates and surface kinetics, i.e., different growth speeds on any given surface. Both phenomena depend critically on the supersaturation. We unveil the MOVPE growth mechanism, which we describe through a sequence of different growth steps. A first nucleation layer, composed of coalesced 3D-β islands, is always present because of the high lattice misfit between Ga_2_O_3_ and the α-Al_2_O_3_ or GaN substrate.

During the subsequent growth of Ga_2_O_3_, the nucleation layer partly acts as a template, the properties of which are strongly influenced by the type of underlying substrate. The further development of the κ or β phase is then mainly driven by two mechanisms: the different residual strain in the β nucleation layer and the different interface energy between the nucleation layer itself and the κ phase.

In addition, our theoretical modeling suggests that the lateral step energy of 2D-κ islands might be smaller than that for 2D-β islands, an issue that may require further experimental investigations.

The results presented in this paper provide insightful information on the role of the interlayer and on the effect of strain in the stabilization of different phases of Ga_2_O_3_. This should allow for advanced engineering of the interface between the substrate and epitaxial layer to improve its crystalline quality and increase the performances of devices based on this wide-band-gap material.

## Supplementary Material



## Notes

Additional information and data on the MBE layer (Section 1), XRD analysis (Section 2), TEM data (Section 3), FEM calculations (Section 4), calculated supersaturation values (Section 5), nucleation curves for the α-Ga_2_O_3_ wetting layer (Section 6), and mismatch of κ- and β-Ga_2_O_3_ and their interface energy in the case of the GaN substrate (Section 7) (PDF)
