# Supplementary material for: Supersaturation-Dependent Competition between β and κ Phases in the MOVPE Growth of Ga2O3 on Al2O3 (0001) and GaN (0001) Substrates
Source: ACS Appl Mater Interfaces. 2025 Oct 31;17(45):62261–76. doi: 10.1021/acsami.5c13401 (PMC12616610; doi:10.1021/acsami.5c13401)
Supplement: Supplementary file 1 [file am5c13401_si_001.pdf]

Supporting Information for

Supersaturation-dependent competition between β and κ phases in the MOVPE growth of Ga_2O_3 on Al_2O_3 (0001) and GaN (0001) substrates

L. Seravalli[1*], A. Ugolotti[5*], R. Bergamaschini[5*], M. Bosi[1*], I. Cora[4 *], F. Mezzadri[3,1], P. Mazzolini[2,1], L. Cademartiri[3], I. Bertoni[5], Zs. Fogarassy[4], B. Pécz[4], O. Bierwagen[6], A. Ardenghi[6], S. Leone[7], L. Nasi[1], L. Miglio[5], and R. Fornari[2,1]

[1] *CNR - Inst. of Materials for Electronics and Magnetism, 43124 Parma, Italy*
[2] *University of Parma, Dept. of Mathematical, Physical and Computer Sciences, 43124 Parma, Italy*
[3] *University of Parma, Dept. of Chem., Life Sciences, Environmental Sustainability, 43124 Parma, Italy*
[4] *HUN_REN Centre for Energy Research, Institute for Technical Physics and Materials Science, 1121 Budapest, Hungary*
[5] *University of Milano-Bicocca, Dept. of Materials Science, 20125 Milano, Italy.*
[6] *Paul-Drude-Institut für Festkörperelektronik, Leibniz-Institut im Forschungsverbund, 10117 Berlin, Germany*
[7] *Fraunhofer Institute for Applied Solid State Physics (IAF), 79108 Freiburg, Germany*

luca.seravalli@cnr.it, matteo.bosi@cnr.it

Section 1: MBE layer properties

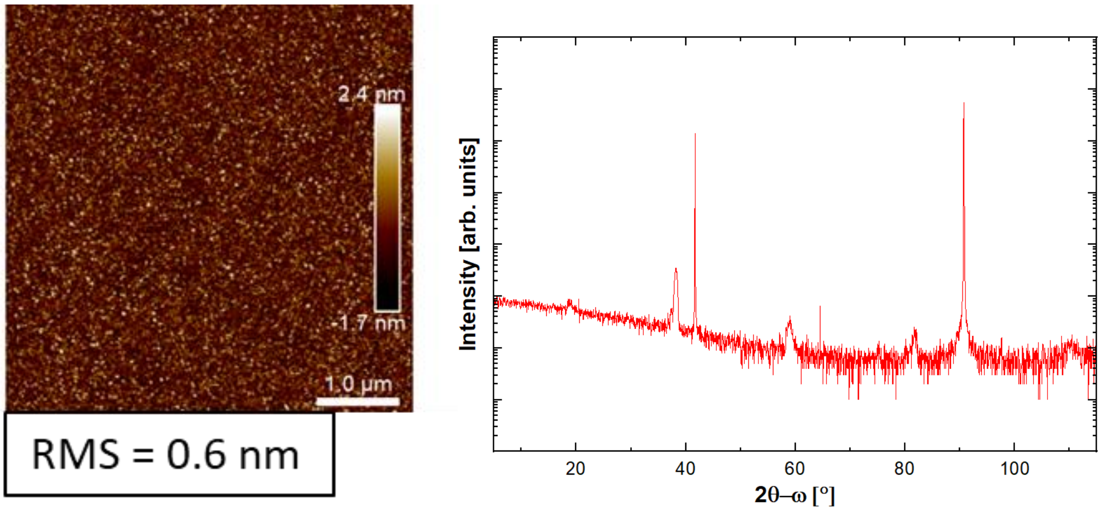

Figure S1: *(Left) AFM micrograph of the MBE layer with derived value of RMS roughness. (Right) XRD pattern of the MBE layer.*

Section 2: Advanced XRD characterization.

An advanced analysis of XRD data was performed, allowing to obtain lattice parameters for κ-Ga_2O_3. In the table we report the derived values of the lattice parameters, alongside relevant growth conditions and the fraction of β-Ga_2O_3, as derived by XRD analysis. Errors in thicknesses and Growth Rate are ± 10 %.

Table S1

sample position	*substrate*	*thickness (nm)*	*GR (nm/min)*	*Beta Fraction (%)*	*a*	*b*	*c*	*Volume*
**A – 1**	**sapphire**	**2350**	**19,6**	*0*	5,051572	8,691694	9,25800	406,49
A – 1.5	sapphire				5,047434	8,675396	9,25640	405,32
A – 2	sapphire	1600	13,3	*30*	5,026826	8,670036	9,29365	405,04
A – 2.5	sapphire				5,04733	8,640284	9,29280	405,26
A – 3	sapphire	1090	9,1	*50*	5,033106	8,655836	9,31140	405,66
A – 4	sapphire	830	6,9					
A – 5	sapphire	570	4,8	*100*				
**B – 1**	**GaN**	**2350**	**19,6**	*0*	5,056073	8,68842	9,25608	406,613
B – 2	GaN	**1600**	**13.3**	*10*	5,04594	8,672736	9,2564	405,0796
B – 2.5	GaN				5,041883	8,677289	9,279198	405,9638
B – 3	GaN	**1090**	**9.1**	*30*	5,04409	8,683324	9,278724	406,4032
B – 4	GaN	830	6,9	*40*				
B – 5	GaN	570	4,8	*50*				
**C – 1**	**sapphire**	**410**	**20,5**	*0*	5,039188	8,694666	9,25542	405,5175
C - 1.5	sapphire				5,051228	8,670569	9,25852	405,4956

C - 2	sapphire	250	12,5		5,030203	8,691338	9,26188	404,9219
C - 3	sapphire	180	9,0		5,056525	8,599424	9,288	403,872

Our method is essentially based on least square fitting of unit cell parameters based on the limit information obtainable from the XRD of oriented thin films, with an error that can be estimated in the 1% range.

The lattice parameters measured here with this novel method agree well (less than 1% difference) for the pure κ-Ga_2O_3 material (point 1 for all samples) for values reported in literature by Kneiss et al.[1] and by Cora et al. [2] From the XRD analysis an effect of the presence of β-Ga_2O_3 on the lattice of κ-Ga_2O_3 can be evidenced: the c lattice parameter is increased, while the a and b are reduced, hinting to a distortion of the orthorombic lattice induced by the presence of the material with a different phase. If one compares the values for the samples grown on c-sapphire and GaN, despite the latter having a richer composition of κ-Ga_2O_3, it appears that the same lattice deformation occurs, although by a lesser amount for the same amount of β-Ga_2O_3 (comparison of points 669-2 and 672-3).

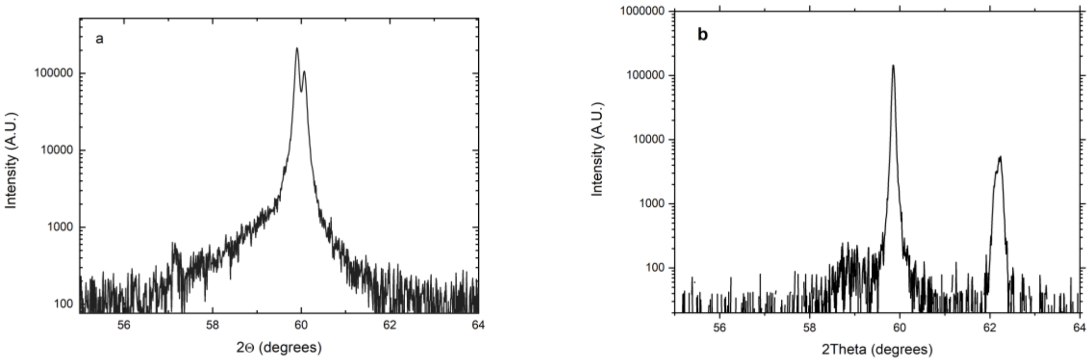

Figure S2: *XRD patterns of samples C (a) and D (b) grown on c-sapphire and β-Ga_2O_3, respectively.*

In Figure S1 (b) the additional peak at 62.2° can be attributed to the probable presence of non-epitaxial κ-Ga_2O_3 crystals embedded in the (001) epitaxial matrix

Section 3: TEM Data

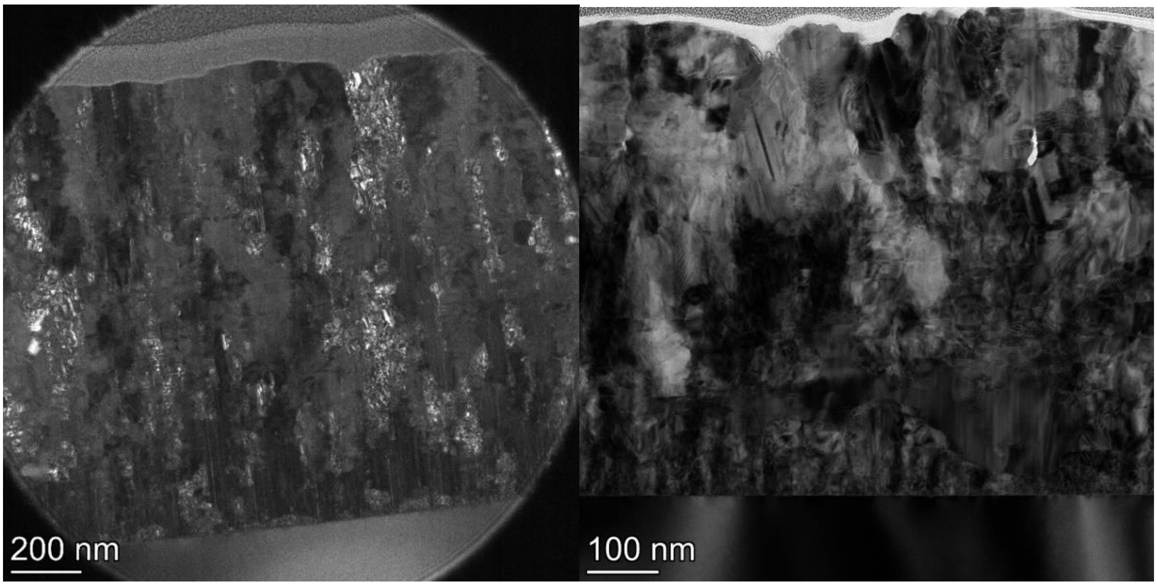

Fig.S3 *Overview TEM Bright Field image of the sample grown on c-sapphire (left) and on GaN (right).*

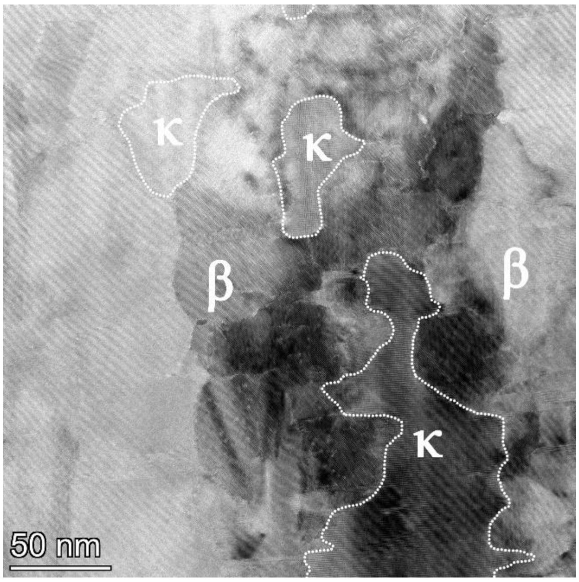

Fig.S4 *Overview TEM Bright Field image of the sample A*

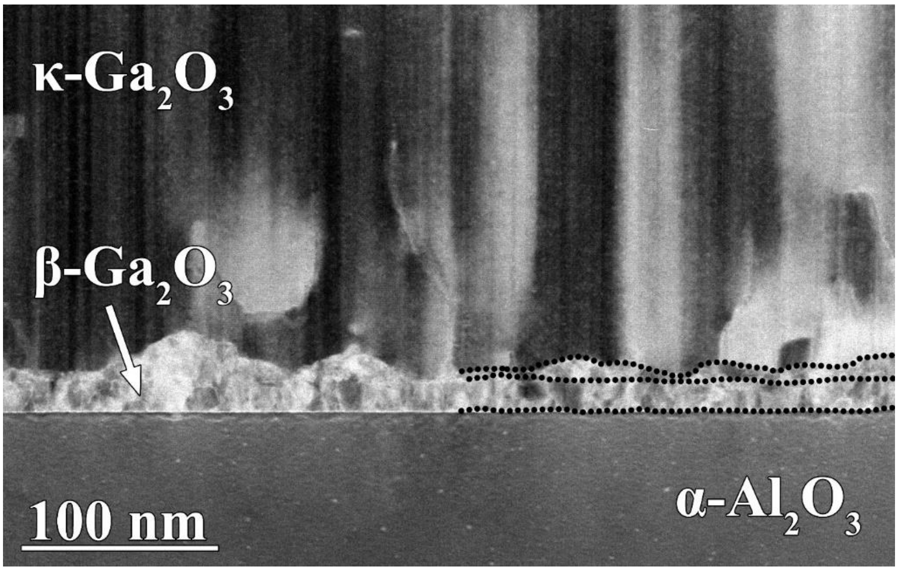

Figure S5. *Bright field TEM image from the interface area between κ-Ga_2O_3 and β-Ga_2O_3/α-Al_2O_3 substrate. Dotted lines show the boundaries between κ-Ga_2O_3, transitional layer, β-Ga_2O_3 and α-Al_2O_3 substrate. The thickness of the β-Ga_2O_3 substrate is approximately 16+/-2 nm, while the transitional layer is 1-30 nm thick.*

TEM investigations on this sample revealed that the thickness of the MBE-grown β-Ga_2O_3 template is approximately 16 ± 2 nm. The template is strongly textured, with the dominant surface being the (20-1)β || (001)α-Al2O3. A transitional layer 1-30 nm thick is grown on the top of the β template, which mainly consists of textured β.

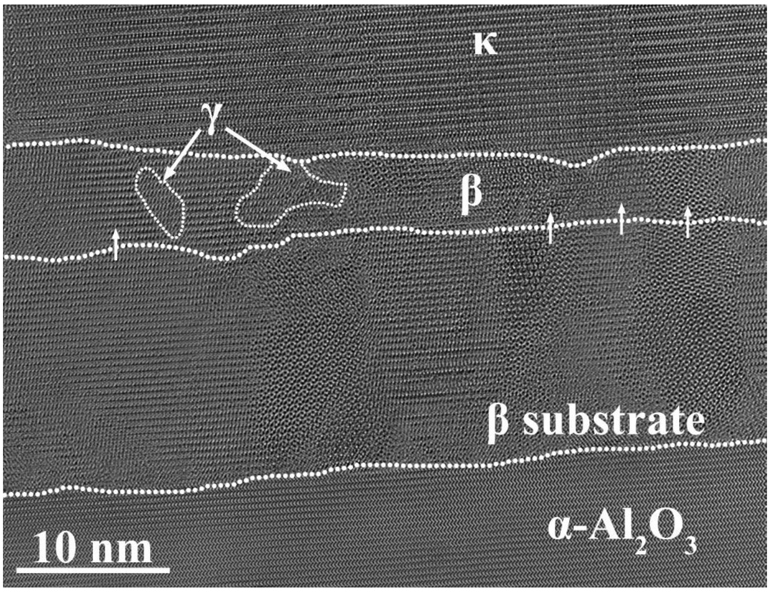

Figure S6: *HRTEM image from the interface area between κ-Ga_2O_3 and β-Ga_2O_3/α-Al_2O_3 substrate. Larger dotted lines show the boundaries between κ-Ga2O3,*

transitional layer, β-Ga_2O_3 and α-Al_2O_3 substrate. White arrow show that the β grain of the transitional layer follow the orientation of the underlying β grain of the substrate.

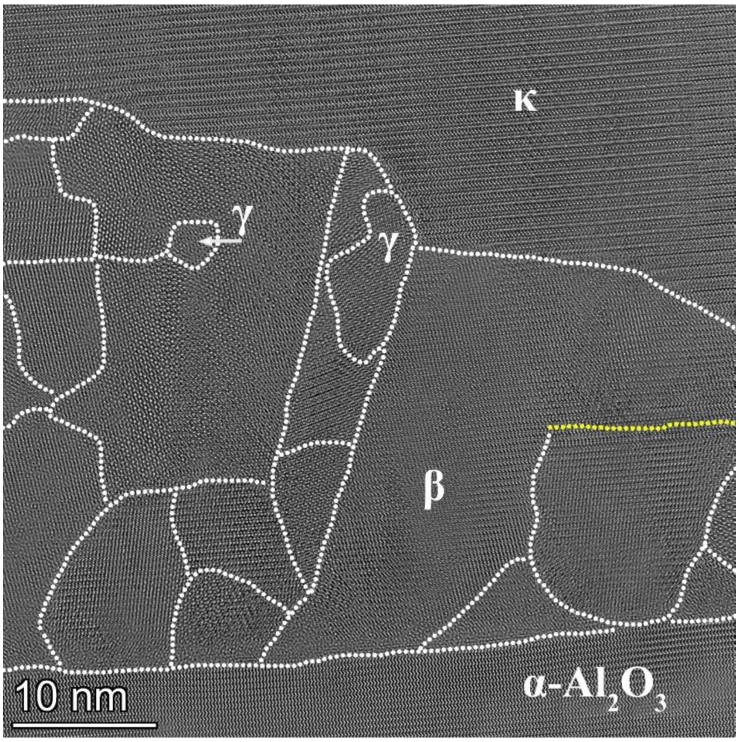

***Figure S7**: HRTEM image from the interface area between κ-Ga_2O_3 and β-Ga_2O_3/α-Al_2O_3 substrate. The white dotted lines show the boundaries of differently oriented β-grains of the β-Ga_2O_3 and the interlayer. The yellow dotted line shows the boundary between the β-Ga_2O_3 substrate and the interlayer.*

***Table S2:** FFT spots identified in the HRTEM images for the different crystals and their corresponding Miller plane distances d. The apparent misfit strain (ε_{xx} and ε_{yy}) expected from the GPA is reported as $(d_{sub} - d_{epi})/d_{sub}$. For β-$Ga_2O_3$, the rotational domains have no symmetric structure and the results for two observed domain are reported. Errors for calculated strain can be estimated to be within a ± 2 % range.*

on Al_2O_3						
	in-plane			*out-of-plane*		
	(hkl)	d (Å)	ε_{xx} (%)	(hkl)	d (Å)	ε_{yy} (%)
substrate	110	2.3800	0.00	*006*	*2.1657*	*0.00*
κ	*200*	2.5231	6.02	*004*	*2.3208*	*7.16*

	{-130}	2.5148	5.66			
β-[010]	401*	2.4504	*2.96*	*40-2*	*2.3414*	*8.11*
β-[132]	*1-11**	*2.5817*	*8.47*			
on GaN						
	in-plane			*out-of-plane*		
	(hkl)	d (Å)	ε_{xx} (%)	(hkl)	d (Å)	ε_{yy} (%)
substrate	110	2.7550	0.00	*002*	*2.5920*	*0.00*
κ	*200*	2.5231	-8.42	*004*	*2.3208*	-10.46
	{-130}	2.5148	-8.72			
β-[010]	401*	2.4504	-11.06	*40-2*	*2.3414*	-9.67
β-[132]	*1-11**	*2.5817*	-6.29			
* projected onto the x axis of the reciprocal space						

Section 4: Residual elastic energy in 3D islands of β-, κ-Ga_2O_3: FEM calculations

The initial shape of the islands has been constructed based on the experimental AFM (taken in contact mode on a sample grown in similar conditions but by lower growth rate – Figure S7a and S7b) and SEM data (taken on a sample grown under similar conditions but with higher growth rate), as shown in Figure S7a, S7b, and S7e, from which we also estimated the aspect ratio (width/height) of the islands. Figure S7c, f reports a sketch of the actual shape given in input to finite elements method (FEM) calculations. In case of 3D-β islands, the surface energies and area/volume geometric factors have been estimated from a Wulff shape through the Winterbottom construction where the interface energy has been tuned to fit the desired aspect ratio (Figure S7d). The required surface energies were taken as (11-2A): 62.4 meV/Å^2 , [3] (113A): 65.4 meV/Å^2 , [3] (100A): 37.0 meV/Å^2, [4] (110, 4Ga+2O): 75.8 meV/Å^2. [5] In the case of the 3D-κ islands, we build the model including the rotational domains as described Ref. [6] The energy surface we calculated for the lateral (130) facets is shown in Figure S8. Details on the computational setup required to perform such density functional theory (DFT) calculations are reported elsewhere. [4,7] The Al_2O_3 and Ga_2O_3 elastic constants where calculated for the oriented crystal structures by DFT, with the same setup described in Ref. [4,7]; their values are reported in Table S3. Through FEM calculations we evaluated the fraction of the elastic energy density retained after

relaxation by the island-plus-substrate system, therefore including the substrate's deformation cost. Then, such result was used to scale the elastic energy density of a fully strained 2D film calculated through DFT.

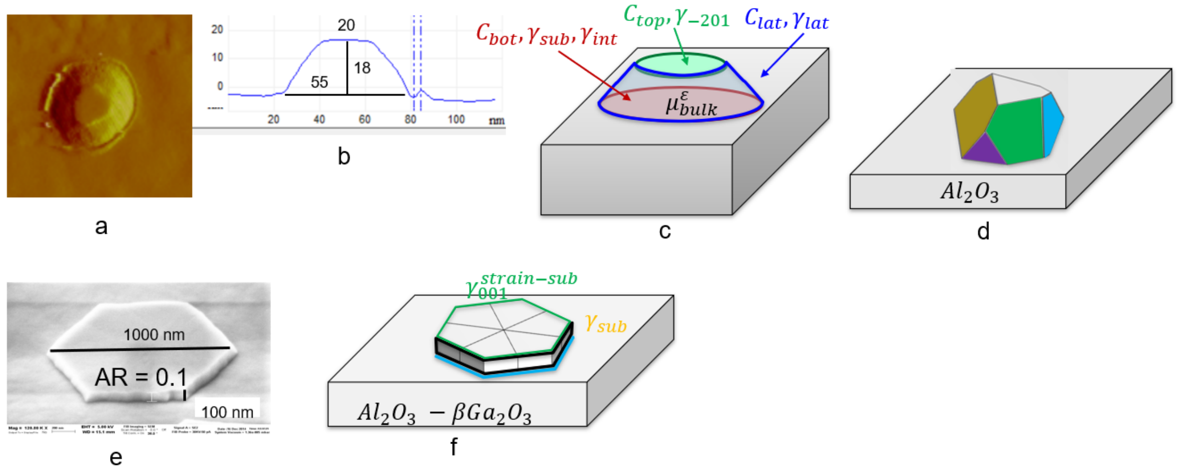

Figure S8*: comparison between the 3D-β (a-d) and 3D-κ (e-f) islands observed through the SEM. The experimental images (a, b, e) are then converted into initial guesses for the FEM models (c, f). Wulff shapes made with Winterbottom construction (d, f) are then considered for the calculation of the geometrical factors needed by our nucleation models.*

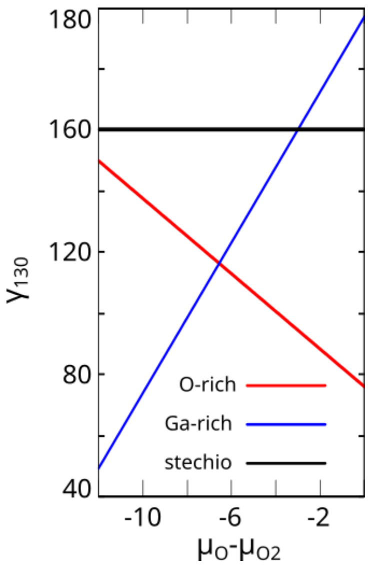

Figure S9*: surface energies of the (130) κ-Ga_2O_3 surface, calculated for stochiometric and non-stochiometric terminations.*

Table S3: *values of the thermodynamic and elastic parameters included in the nucleation model considered in this work.*

<u>2D islands</u>: energy difference from the most stable unstrained phasedif μ_{chem} (meV/f.u.), *elastic energy density* $\Delta\mu_{2D}^{\varepsilon}$ (meV/f.u.), *volume per formula unit* ν *($Å^3$/f.u.), height of an asymmetric mononalyer* h *(Å), surface energy* γ_{top} *(meV/$Å^2$) of the top facet of the island and interface energy* γ_{int} *(meV/$Å^2$). Values calculated for different strain conditions, namely matching the lattice parameters of Al_2O_3 or the fully/partly (at 50%) relaxed β-Ga_2O_3 template ($\varepsilon_0/\varepsilon_{50}$) are reported.(*) the different values correspond to a different* α-*Ga_2O_3 film thickness: single layer or thick film, respectively.*

	2D-α	2D-β	2D-κ
μ_{chem}	45	0	63
$\Delta\mu_{2D}^{\varepsilon}$@$Al_2O_3$	309	540	314
$\Delta\mu_{2D}^{\varepsilon}$@β-$Ga_2O_3$ $\varepsilon_0/\varepsilon_{50}$	-	0/135	69/74
ν @Al_2O_3	45.8	48.6	48.1
ν @β-Ga_2O_3 $\varepsilon_0/\varepsilon_{50}$	-	52.8/51.75	51.8/50.0
h @Al_2O_3	2.32	4.93	4.88
h @β-Ga_2O_3 $\varepsilon_0/\varepsilon_{50}$	-	4.69/4.81	4.60/4.74
γ_{top} @Al_2O_3	93.6/91.0*	85	86
γ_{top} @β-Ga_2O_3 $\varepsilon_0/\varepsilon_{50}$	-	58/71	82/84
γ_{int} @Al_2O_3	-3	53	27
γ_{int} @β-Ga_2O_3 $\varepsilon_0/\varepsilon_{50}/\varepsilon_{100}$	-	0	20.0/17.5/15.0
γ_{int} @α-Ga_2O_3	0	52.0	30.0
3D-β islands			
$\Delta\mu_{2D}^{\varepsilon}$@$Al_2O_3$ (meV/f.u.meV/)	178.2 (32.6%)		
γ_{-201} (meV/$Å^2$) $\varepsilon_0/\varepsilon_{50}/\varepsilon_{100}$	58.0/54.0/50.0		
γ_{lat} (meV/$Å^2$)	54.1		
γ_{int}^{Al2O3}, $\gamma_{int}^{\alpha-Ga2O3}$ (meV/$Å^2$)	53.0, 52.0		
$\gamma_{sub}^{\alpha-Ga2O3}$ (meV/$Å^2$)	93.6/91.0*		
γ_{sub}^{Al2O3} (meV/$Å^2$)	113.0		
3D-κ islands			

$\Delta\mu_{2D}^{\varepsilon}$@$Al_2O_3$ (meV/f.u.)	152.1 (48.5%)
$\Delta\mu_{2D}^{\varepsilon}$@β-$Ga_2O_3$ (meV/f.u.) ε_0	116.8 (16.5%)
$\Delta\mu_{2D}^{\varepsilon}$@β-$Ga_2O_3$ (meV/f.u.) ε_{50}	98.9 (10.8%)
$\Delta\mu_{2D}^{\varepsilon}$@β-$Ga_2O_3$ (meV/f.u.) ε_{100}	190.3 (39.9%)
$\gamma_{001-reconstructed}^{@Al2O3}$ (meV/Å^2)	86.0
$\gamma_{001-reconstructed}^{@\beta-Ga2O3}$ (meV/Å^2) $\varepsilon_0/\varepsilon_{50}$	82.0/84.0
γ_{130} (meV/Å^2)	85.0
$\gamma_{int}^{Al2O3}, \gamma_{int}^{\alpha-Ga2O3}$ (meV/Å^2)	27.0, 30.0
$\gamma_{int}^{\beta-Ga2O3}$ (meV/Å^2) $\varepsilon_0/\varepsilon_{50}/\varepsilon_{100}$	20.0/17.5/15.0
$\gamma_{sub}^{\beta-Ga2O3}$ (meV/Å^2) $\varepsilon_0/\varepsilon_{50}/\varepsilon_{100}$	58.0/71.0/85.0
$\gamma_{sub}^{\alpha-Ga2O3}$ (meV/Å^2)	93.6/91.0*
γ_{sub}^{Al2O3} (meV/Å^2)	113.0

Bulk elastic constants C_{ij} Voigt notation (GPa)					
**(-201) β-Ga_2O_3**					
329	100	89	-20	0	0
100	263	129	41	0	0
89	129	276	15	0	0
-20	41	15	69	0	0
0	0	0	0	58	-23
0	0	0	0	-23	87
**(001) κ-Ga_2O_3**					
320	156	132	0	0	0
156	281	136	0	0	0
131	136	276	0	0	0
0	0	0	74	0	0
0	0	0	0	50	0
0	0	0	0	0	93
**(001) α-Al_2O_3**					
470	157	118	19	0	0
157	470	118	-19	0	0
118	118	467	0	0	0
19	-19	0	143	0	0
0	0	0	0	143	19

0	0	0	0	-19	157

Section 5: calculated supersaturation values

The experimental supersaturation values have been estimated through the following formula:

$$\Delta\mu^{expt} = k_B T ln\left(\frac{p^{growth}}{p_{eq}}\right) = k_B T ln\left[\left(\frac{F_{TMG}^{nominal}}{F_{TMG}^{minimum}}\right)\left(\frac{GR^{pos-growth}}{GR^{outlet}}\right)\right] \quad (S1)$$

The resulting values, using the nominal pressures and growth rates previously measured in Ref. [8], are collected in Table S4.

Table S4: *supersaturation values, as a function of the nominal TMG flux and position on the sample, expressed in meV/f.u., calculated at different experimental conditions.*

	T=610 °C			T=650°C		
TMG flux	Inlet	Center	Outlet	Inlet	Center	Outlet
High (F_{TMG})	0.29	0.26	0.22	0.31	0.27	0.23
Intermediate (1/5 F_{TMG})	0.18	0.13	0.10	0.18	0.4	0.10
Low (1/18 F_{TMG})	0.16	0.11	0.00	0.17	0.11	0.0

Section 6: nucleation curves for the case of the α-Ga_2O_3 wetting layer

Assuming the growth of one single layer of α-Ga_2O_3 fully strained to match the underlying (0001)-Al_2O_3 substrate, the resulting curves for the critical nuclei barriers and nucleation rates are collected in Figure S9. The 2D-α islands are now penalized with respect to the 3D-β ones because of the reduced energy gain in covering the substrate (α-Ga_2O_3) free energy, which is lower than that of the (001)-Al_2O_3. Therefore, at those growth conditions, and for the values of the step-energy λ which promote the initial growth of 2D-α on sapphire, the nucleation of the same islands is now hindered, allowing for the growth of 3D-β ones. The growth of thicker α-Ga_2O_3 layers, observed with other deposition techniques, such as MIST-CVD [9] or MOVPE with the addition of

HCl, [10] must be justified in terms of kinetic effects. Previous literature studies[7,9] already discussed the occurrence of “epitaxial phase-locking”, i.e. the tendency of prosecuting a planar growth of the same phase of the substrate even if it is not the most stable one. This means that for c-sapphire substrate phase-locking promotes the 2D nucleation of α layers rather than switching to the thermodynamically more convenient 3D β islands.

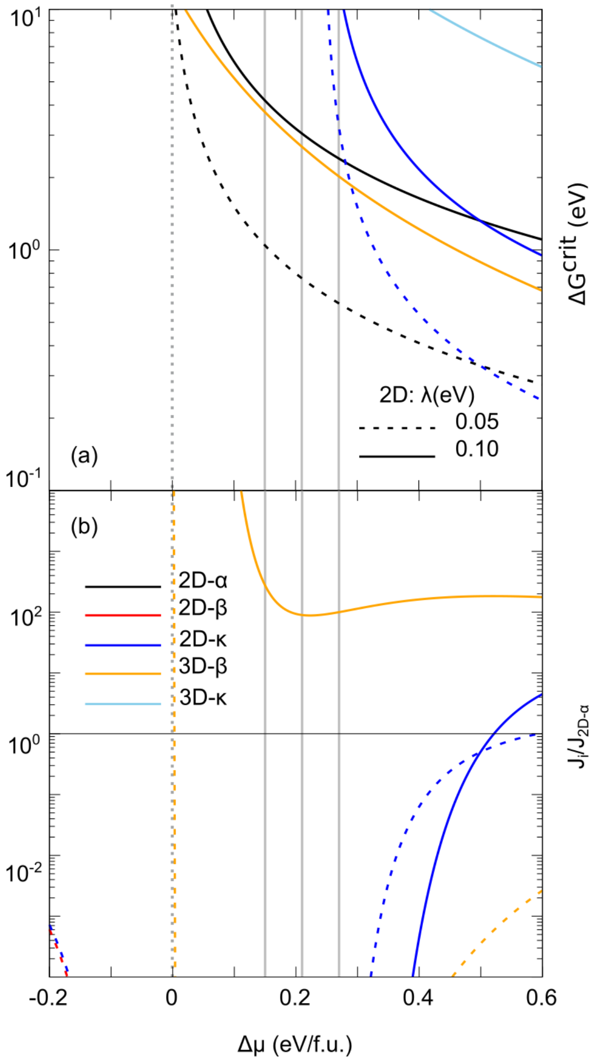

Figure S10: *nucleation barriers (a) and ratio of the nucleation rates vs 2D-α (b) for the islands of Ga_2O_3 polymorphs calculated on a single α-Ga_2O_3 layer as substrate, strained to match the lattice of the (001) sapphire surface.*

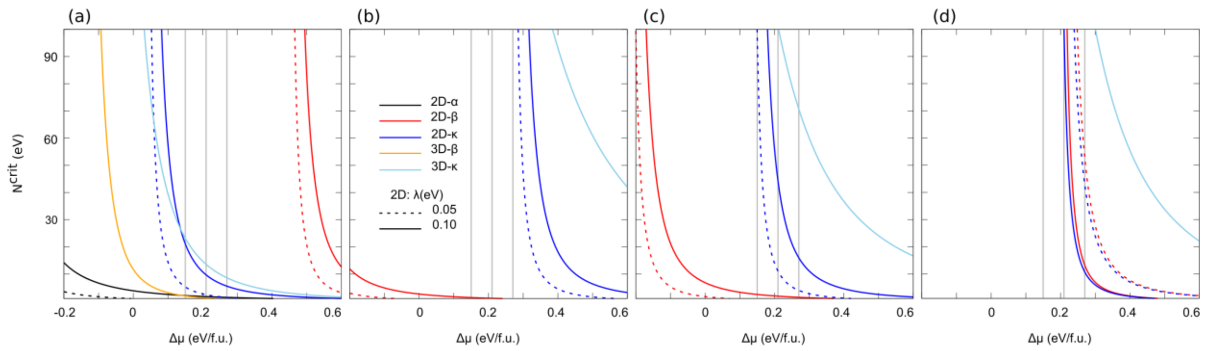

Figure S11: *plot of the size of the critical nuclei calculated for the different island shapes of different Ga_2O_3 polymorphs on (a) Al_2O_3, or β-Ga_2O_3 (b) fully relaxed, (c) 50% residual strain and (d) fully strained vs Al_2O_3. The vertical gray lines indicate the supersaturation values we estimated for sample A at inlet, center or outlet positions.*

Section 7: mismatch of κ- and β-Ga_2O_3 and their interface energy in the case of GaN substrate.

The identification of the epitaxial relationship between a film made of κ-Ga_2O_3 and a β-Ga_2O_3 substrate is a crucial step, since it defines the thermodynamic parameters within the model of nucleation. Its calculation follows two possible paradigms: i) maintaining a shared layer of O atoms at the interface or ii) minimizing the misfit strain between the two crystals. Indeed, the former constitutes a more constrained approach, yet it is expected to benefit from the minimal interface energy (15-20 meV/Å^2) previously calculated in Ref. [7]

Assuming conventional, rectangular in-plane lattices along the growth planes, i.e β-(-201) and κ-(001), a sketch of these two different approaches are shown in Figure S13.

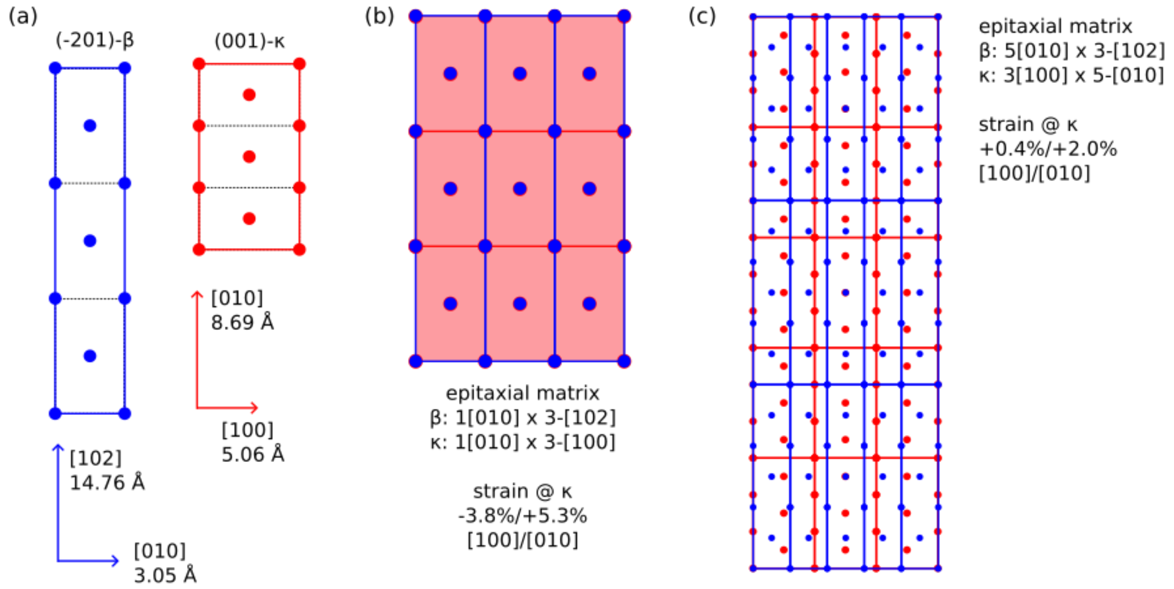

Figure S12*: comparison of the lattice parameters of fully-relaxed β- and κ-Ga_2O_3 (panel a) and epitaxial relationships between the two, calculated with two different approaches: matching the positions of a common layer of O atoms at the interface (panel b) or through the commensurate pattern which minimizes the misfit strain (panel c).*

Keeping these epitaxial relationships between the Ga_2O_3 phases, we now take into account the GaN substrate. In this case, the structure has no oxygen, yet thanks to the tetragonal coordination of Ga or N atoms at the surface exposing a single dangling bond each, we can envision also in this case the presence of a layer of O atoms at the interface, facilitating the bonding of the β-Ga_2O_3 film to the GaN surface. Therefore, we can study the epitaxial relationship between these two layers as we did for κ- on β-Ga_2O_3; a sketch of the different commensurate supercells is reported in Figure S14.

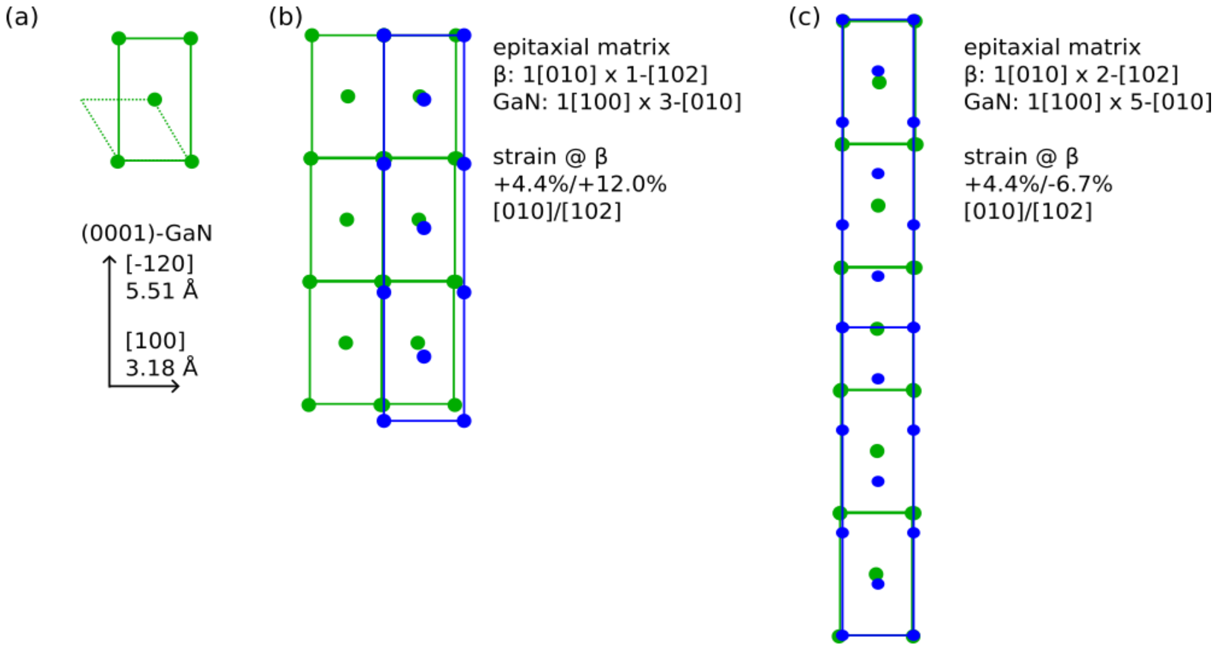

***Figure S13**: lattice parameters of fully-relaxed (0001)-GaN substrate (panel a) and the epitaxial relationships with β-Ga_2O_3, calculated with two different approaches: matching the positions of a common layer of O atoms at the interface (panel b) or through the commensurate pattern which minimizes the misfit strain (panel c).*

These results provide two values for the maximum strain of an ideal 2D of β-Ga_2O_3. Therefore, in order to evaluate the role of different degrees of strain release in such 2D-β substrates for the subsequent growth of 2D-κ films, we calculated the bulk elastic contributions as a function of the residual strain of the β phase, as shown in Figure S15.

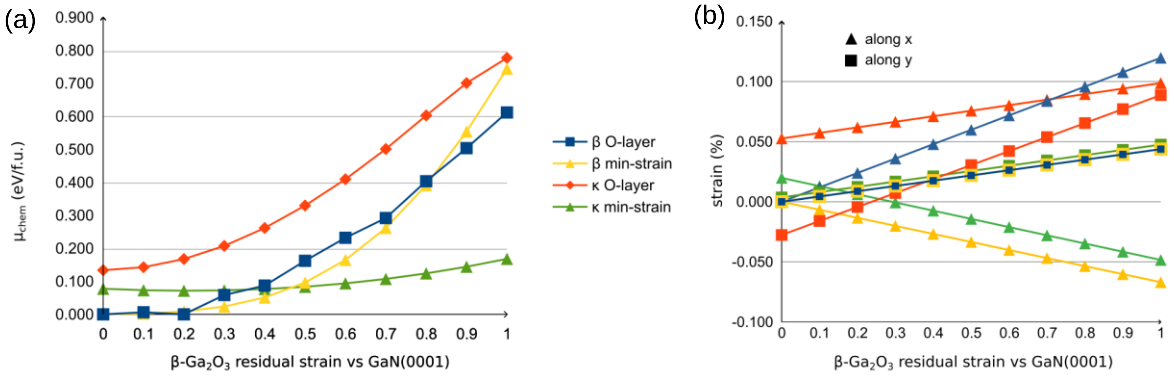

***Figure S14**: comparison between the chemical potential (including elastic and chemical contributions) (panel a) and the misfit strain values (panel b) of oriented bulks of β- and κ-Ga_2O_3 calculated with different epitaxial relationships, as a function of the*

residual strain of the β film (in the case of GaN substrate). The x direction is defined as the [010] for the β phase and as the [100] direction for the κ phase.

It can be observed that, while the chemical potential of β bulk doesn't depend much on how we do estimate its match with the GaN substrate, the elastic contribution of κ-Ga_2O_3 is drastically lower if we tend to minimize the lattice strain overall, rather than preserving the O interface with the β substrate. Indeed, our nucleation model should predict the formation of no 2D/3D-κ islands in the former case with such values of elastic energy. However, the latter case requires the re-evaluation of the inteface energy, because of the presence of potential Ga-O broken bonds. In order to obtain a fast, yet realistic, estimate of the order of magnitude of the interface energy of a κ-Ga_2O_3 on a fully-relaxed β-Ga_2O_3 substrate,we performed such calculation using state-of-the-art machine learned GAP force fields, rather than DFT. We first reproduced the "ideal" interface with the share O layer and we compared it with that we calculated in Ref.[7] through DFT (44 meV/Å^2 vs 21 meV/Å^2), providing a numeric scale coefficient between the two methods. The interface energy calculated for the optimized structure is as low as 52 meV/Å^2, equivalent to 25 meV/Å^2 in a DFT calculation.

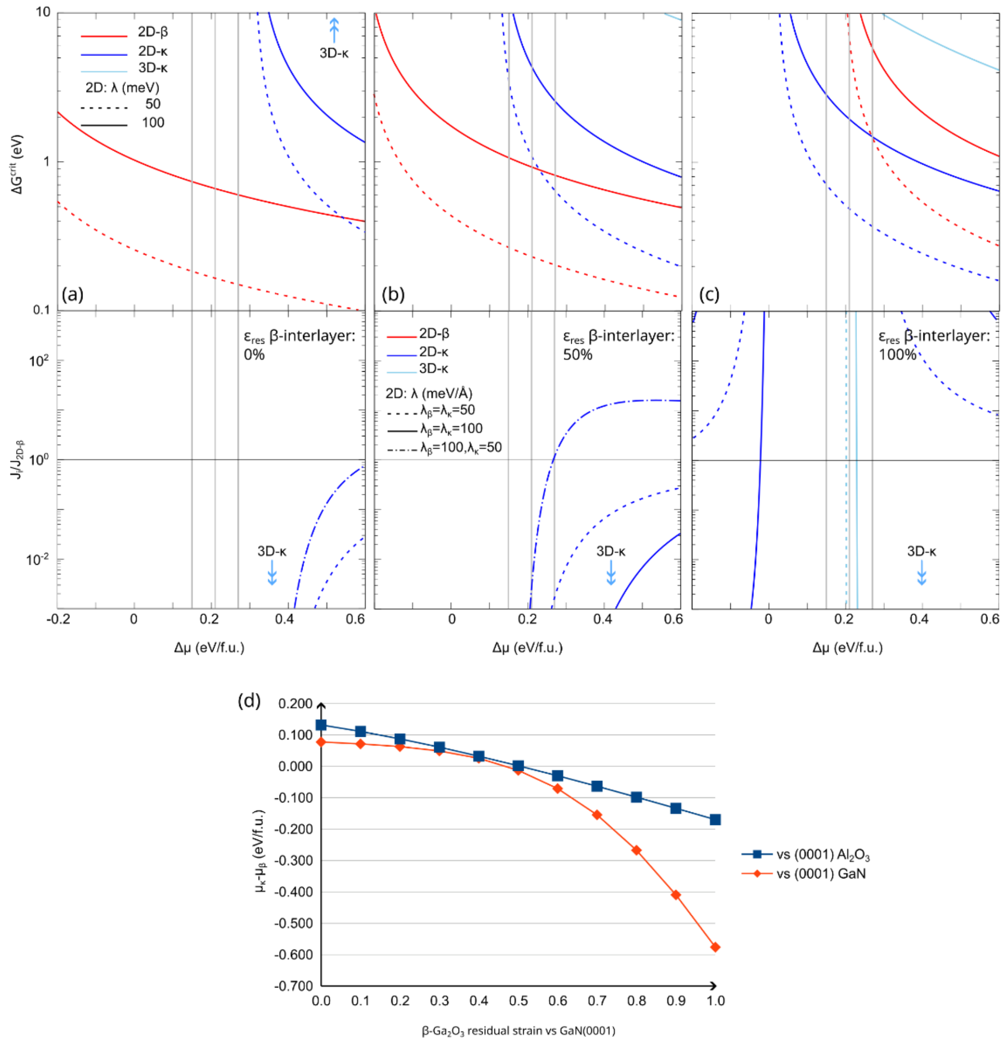

***Figure S15**: comparison between the nucleation barrier of 2D-β, 2D-κ and 3D-κ islands on a β-Ga_2O_3 substrate with different strain state: fully strained (panels a), fully relaxed (panels c) or at 50% residual strain (panels b) vs the GaN lattice. The top panels report the barrier of the critical nuclei, the lower panels report the nucleation rate of κ islands vs 2D-β ones. Panel d shows the difference in chemical potential among the two Ga_2O_3 phases between the two different substrates.The elastic energy in this case has been calculated accounting for an epitaxial matrix which minimizes the overall lattice misfit (see below).*

References

(1) Kneiß, M.; Hassa, A.; Splith, D.; Sturm, C.; Von Wenckstern, H.; Schultz, T.; Koch, N.; Lorenz, M.; Grundmann, M. Tin-Assisted Heteroepitaxial PLD-Growth of κ -Ga 2 O 3 Thin Films with High Crystalline Quality. *APL Mater* **2019**, *7* (2), 022516. https://doi.org/10.1063/1.5054378.

(2) Cora, I.; Mezzadri, F.; Boschi, F.; Bosi, M.; Čaplovičová, M.; Calestani, G.; Dódony, I.; Pécz, B.; Fornari, R. The Real Structure of ε-Ga 2 O 3 and Its Relation to κ-Phase. *CrystEngComm* **2017**, *19* (11), 1509–1516. https://doi.org/10.1039/c7ce00123a.
(3) Hinuma, Y.; Kamachi, T.; Hamamoto, N.; Takao, M.; Toyao, T.; Shimizu, K. Surface Oxygen Vacancy Formation Energy Calculations in 34 Orientations of β-Ga_2O_3 and θ-Al_2O_3. *The Journal of Physical Chemistry C* **2020**, *124* (19), 10509–10522. https://doi.org/10.1021/acs.jpcc.0c00994.
(4) Bertoni, I.; Ugolotti, A.; Scalise, E.; Miglio, L. Surface and Volume Energies of α-, β-, and κ-Ga_2O_3 under Epitaxial Strain Induced by a Sapphire Substrate. *J Mater Chem C Mater* **2024**, *12* (5), 1820–1832. https://doi.org/10.1039/D3TC04284G.
(5) Wang, M.; Mu, S.; Van de Walle, C. G. Surface Reconstructions on Bare and Hydrogenated β−Ga2O3 Surfaces: Implications for Growth. *Phys Rev Mater* **2023**, *7* (6), 064603. https://doi.org/10.1103/PhysRevMaterials.7.064603.
(6) Oshima, Y.; Kawara, K.; Oshima, T.; Shinohe, T. In-Plane Orientation Control of (001) κ-Ga_2O_3 by Epitaxial Lateral Overgrowth through a Geometrical Natural Selection Mechanism. *Jpn J Appl Phys* **2020**, *59* (11), 115501. https://doi.org/10.35848/1347-4065/abbc57.
(7) Bertoni, I.; Ugolotti, A.; Scalise, E.; Bergamaschini, R.; Miglio, L. Interface Energies of Ga_2O_3 Phases with the Sapphire Substrate and the Phase-Locked Epitaxy of Metastable Structures Explained. *J Mater Chem C Mater* **2025**, *13* (3), 1469–1476. https://doi.org/10.1039/D4TC04307C.
(8) Bosi, M.; Seravalli, L.; Mazzolini, P.; Mezzadri, F.; Fornari, R. Thermodynamic and Kinetic Effects on the Nucleation and Growth of ε/κ- or β-Ga_2O_3 by Metal–Organic Vapor Phase Epitaxy. *Cryst Growth Des* **2021**, *21* (11), 6393–6401. https://doi.org/10.1021/acs.cgd.1c00863.
(9) Kaneko, K.; Kawanowa, H.; Ito, H.; Fujita, S. Evaluation of Misfit Relaxation in α-Ga_2O_3 Epitaxial Growth on α-Al_2O_3 Substrate. *Jpn J Appl Phys* **2012**, *51* (2R), 020201. https://doi.org/10.1143/JJAP.51.020201.
(10) Sun, H.; Li, K.-H.; Castanedo, C. G. T.; Okur, S.; Tompa, G. S.; Salagaj, T.; Lopatin, S.; Genovese, A.; Li, X. HCl Flow-Induced Phase Change of α-, β-, and ε-Ga_2O_3 Films Grown by MOCVD. *Cryst Growth Des* **2018**, *18* (4), 2370–2376. https://doi.org/10.1021/acs.cgd.7b01791.
